# Circulating osteocalcin and incident dementia in the UK Biobank: a prospective cohort and exploratory multi-omics analysis

**DOI:** 10.3389/fnagi.2026.1974960

**Published:** 2026-10-26

**Authors:** Xinxiang Su, Yan Mao, Chao Yang, Hongmei Liao, Hong Zhao, Yingjun Zhang, Wei Wei, Shengxiang Bi, Bo Liu, Xiaoqian Fu, Liang Ren, Hongping Yu, Yihua Yang, Ming Liao

**Affiliations:** 1 Reproductive Medical Center, The First Affiliated Hospital of Guangxi Medical University, Nanning, China; 2 Guilin Medical University, Guilin, China; 3 Department of Experimental Research, Guangxi Medical University Cancer Hospital, Nanning, China; 4 Guangxi Clinical Research Center for Reproductive Medicine, Nanning, China; 5 Department of Toxicology, School of Public Health, Guangxi Medical University, Nanning, Guangxi, China; 6 Key Laboratory of Early Prevention and Treatment for Regional High Frequency Tumor (Guangxi Medical University), Ministry of Education, Nanning, Guangxi, China; 7 Key Cultivated Laboratory of Cancer Molecular Medicine of Guangxi Health Commission, Guangxi Medical University Cancer Hospital, Nanning, Guangxi, China; 8 Guangxi Zhuang Autonomous Region Molecular Medical Engineering Research Center for Cancer, Nanning, Guangxi, China; 9 Department of Epidemiology and Health Statistics, School of Public Health of Guangxi Medical University, Nanning, China

**Keywords:** bone-brain axis, dementia, Osteocalcin, proteomics, UK Biobank

## Abstract

**Background:**

We examined baseline relative circulating osteocalcin (OCN) in relation to incident dementia in UK Biobank.

**Methods:**

The longitudinal cohort comprised 43,942 participants; models used available complete records. Cox models used baseline OCN standardized per SD and follow-up censored at diagnosis, death, or November 27, 2024. Cognitive, MRI, and baseline molecular analyses were exploratory.

**Results:**

In the fully adjusted model, OCN was not clearly associated with all-cause dementia (HR 1.052; 95% CI, 0.988–1.120; *p* = 0.117; *N* = 40,253; 1,095 events). The vascular dementia estimate was nominally positive (HR 1.159; 95% CI, 1.010–1.328; *p* = 0.035), but did not survive Holm correction across three secondary outcomes (adjusted *p* = 0.105) and weakened after excluding baseline stroke. Adding OCN changed the mean fivefold cross-validated C index from 0.84628 to 0.84642 (ΔC = 0.00014). No baseline cognitive association survived Holm correction across four tests. Nominal hippocampal and WMH associations in the larger MRI sample were not evident in matched extensively adjusted samples. Repeated-visit cognitive models did not yield adequately sized multiple classes for interpretable class-membership regressions. Baseline measured-only molecular analyses identified OCN-related correlations, but age- and sex-adjusted BCAA correlations did not survive panel-wise Bonferroni correction.

**Conclusion:**

Baseline relative OCN did not show a clear fully adjusted all-cause dementia association or useful incremental discrimination. Subtype, cognitive, imaging, and molecular findings remain exploratory. These data do not establish a compensatory mechanism or clinical predictive utility.

## 1 Introduction

The projected increase in dementia prevalence highlights the need for prevention and a broader understanding of disease biology (GBD 2019 Dementia Forecasting Collaborators, 2019). The 2022 Alzheimer’s disease drug-development pipeline included diverse treatment targets (Cummings et al., 2022), and the 2020 Lancet Commission reviewed modifiable risks across the life course (Livingston et al., 2020). Physical frailty provides a clinical framework for describing vulnerability in older adults (Fried et al., 2001); it does not by itself establish an endocrine cause of cognitive decline. Experimental and physiological research has also identified endocrine functions of the skeleton, motivating investigation of bone-derived signals beyond their structural roles (Karsenty and Oury, 2012).

The skeleton has endocrine functions (Karsenty and Oury, 2012), and experimental work has examined broader OCN biology (Karsenty, 2023; Moser and van der Eerden, 2018), energy metabolism (Lee et al., 2007), reproduction (Oury et al., 2011), muscle adaptation (Mera et al., 2016), and stress responses (Berger et al., 2019). Mouse studies report effects of OCN on brain development, neurotransmission and memory-related processes, including GPR158 signaling (Oury et al., 2013; Khrimian et al., 2017). These experiments provide biological motivation but do not establish comparable effects of a relative circulating Olink signal in humans.

Human genetic and observational studies have examined OCN in relation to Alzheimer’s disease, cognitive performance, and brain microstructure (Liu et al., 2023; Puig et al., 2016; Bu et al., 2020). Differences in design and measurement limit direct comparison with the present relative plasma assay, and these findings do not establish neuroprotection in this cohort.

Human observations do not show a uniform relationship between higher OCN and adverse outcomes. Plasma and CSF OCN have been examined in relation to AD biomarkers and cognition (Bu et al., 2020), whereas studies in older community-dwelling men and men referred for coronary angiography reported U-shaped OCN–mortality associations, with greater risk at both low and high concentrations (Yeap et al., 2012; Lerchbaum et al., 2013). These outcomes and clinical populations differ from incident dementia in UK Biobank.

Cross-sectional human studies provide heterogeneous evidence concerning OCN and cognitive or neurological phenotypes (Puig et al., 2016; Bu et al., 2020; Bradburn et al., 2016). Schatz et al. (2020) reviewed OCN, ovarian senescence, and brain health, whereas Vijayan et al. (2025) tested undercarboxylated OCN in experimental AD mouse models. Reviews of OCN/GPR158 and bone–brain communication summarize mechanistic proposals rather than independent clinical validation (Li et al., 2025; Chen et al., 2026). Small human samples, sex- and population-specific findings, differing assays, and uncertain temporal direction limit direct comparison. Large-scale plasma proteomic studies provide a rationale for characterizing OCN-related systemic associations (Walker et al., 2021; Walker et al., 2023), without presuming an endocrine decompensation mechanism. The prospective dementia association and descriptive molecular context are therefore evaluated separately here.

The UK Biobank offers linked clinical, genetic, imaging, proteomic, and metabolomic information for exploring OCN-related phenotypes (Bycroft et al., 2018; Sun et al., 2023). We aimed to assess whether baseline relative OCN expression was associated with incident dementia after adjustment for measured covariates, and to explore its associations with cognitive, imaging, proteomic, and metabolomic features. These complementary analyses were intended to characterize associations rather than establish a causal bone-brain mechanism.

## 2 Methods

### 2.1 Study population and sample

UKB is a large-scale prospective cohort study that recruited over 500,000 participants aged 37–73 years from 22 assessment centers across the United Kingdom between 2006 and 2010. The study protocol is available online^1^. This analysis was conducted under UK Biobank application number 190997. The study received ethical approval from the North West Multi-Centre Research Ethics Committee (11/NW/0382), and all participants provided electronic signed informed consent at recruitment.

In the present study, we focused on participants with available proteomic data from the UK Biobank Pharma Proteomics Project (UKB-PPP), which measured plasma protein levels using the Olink Explore 3,072 platform. Because this study constitutes a retrospective analysis of an existing eligible cohort, no formal *a priori* sample-size or statistical power calculation was performed; the analytical sample size was intrinsically determined by the availability of valid Olink data and clinical covariates. Following standard epidemiological protocols, we applied strict inclusion and exclusion criteria. Analytical samples were defined separately for each outcome and its required covariates; completeness across all phenotypes was not required for the primary longitudinal analyses. For the cross-sectional analyses, these eligible participants were included to assess baseline physiological associations. However, for the longitudinal survival analyses evaluating incident dementia risk, to minimize reverse causality and potential confounding from pre-existing neurological damage, we explicitly excluded participants who, at baseline, met any of the following criteria: (1) a prior diagnosis of dementia, Parkinson’s disease, multiple sclerosis, or other severe neurodegenerative and demyelinating disorders (e.g., amyotrophic lateral sclerosis, Huntington’s disease; detailed ICD-10 codes are provided in Supplementary Table S1); or (2) a history of severe head injury or neurotrauma. The longitudinal cohort comprised 43,942 participants. All model-specific sample sizes refer to observed complete records. The cohort flow and analysis families are summarized in Figure 1.

**Figure 1:**
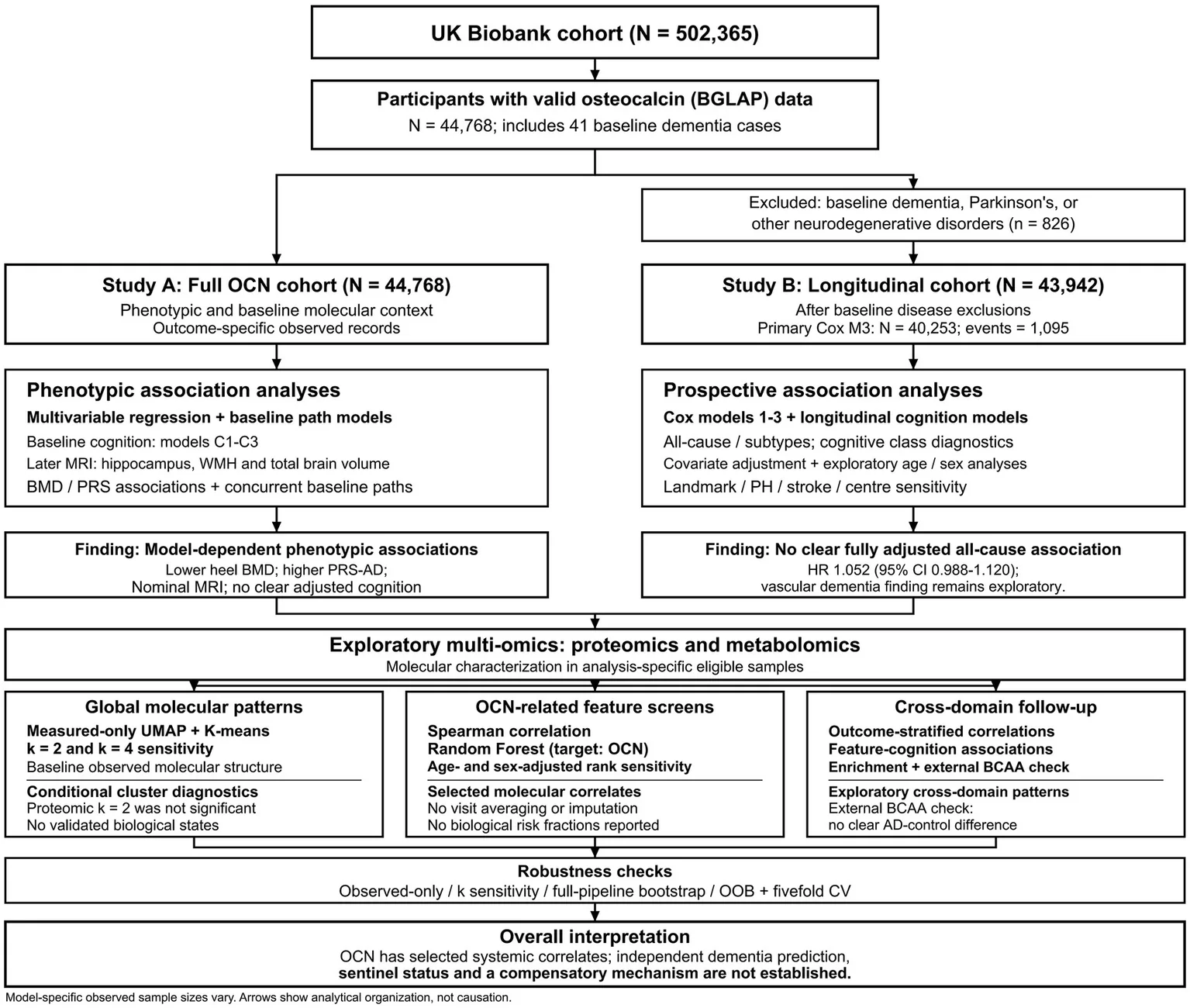
Study population, analytic workflow, and principal findings. The source baseline OCN cohort contains 44,768 participants; the longitudinal cohort contains 43,942 after the study exclusions. The primary Model 3 complete-record sample includes 40,253 participants and 1,095 all-cause dementia events. Cognitive, MRI, and molecular analyses use their outcome-specific observed samples and measurement visits, as specified in Methods and supporting tables. Baseline cognition uses C1–C3; structural MRI uses the later first imaging visit and its separately defined M1–M3 models. Molecular screens use observed baseline pairs, while UMAP, clustering, and Random Forest use the measured complete matrices with the declared feature-availability filter. The diagram distinguishes the primary prospective dementia analysis from exploratory phenotype and molecular analyses. Arrows show the study workflow and do not establish temporal mediation or causal direction. The principal all-cause result and limited incremental discrimination guide interpretation of the exploratory findings.

### 2.2 Primary exposure

Olink BGLAP was measured as Normalized Protein eXpression (NPX) on a log2 scale. Primary Cox and baseline cognitive coefficients are per one SD of baseline OCN, standardized in the available prospective cohort. MRI coefficients remain per one NPX unit for ln(volume); no additional log or *Z* transformation was applied to MRI OCN. Molecular results are Spearman correlations or age- and sex-adjusted partial rank correlations. These quantities and scales are labeled separately.

We refer to the Olink BGLAP signal as relative circulating OCN abundance. NPX is not an absolute concentration. The available assay information and cohort data do not establish isoform-specific detection or the relative antibody response to carboxylated and undercarboxylated OCN. We therefore do not interpret this measurement as an isoform-specific concentration, a validated quantitative total-OCN assay, or a measure of the ucOC/cOC ratio.

### 2.3 Primary outcome

The primary outcome was incident all-cause dementia. Secondary analyses examined Alzheimer’s disease (AD), vascular dementia (VaD), and frontotemporal dementia (FTD). Outcomes were ascertained from linked UK Biobank algorithmic dementia reports and first-recorded ICD-10 diagnoses. AD used G30/F00 and the algorithmic AD report; VaD used F01 and the algorithmic VaD report; FTD used the algorithmic FTD report. The earliest available dementia date was used for ascertainment.

The follow-up period was calculated from the date of attending the assessment center to the date of diagnosis, death, or the end of follow-up (November 27, 2024), whichever occurred first.

### 2.4 Cognitive outcomes

The baseline cognitive analyses used initial-assessment (instance 0) scores in the available incident-dementia cohort, measured concurrently with baseline OCN. Fluid intelligence and numeric memory scores increase with better performance; reaction time and pairs-matching errors increase with worse performance. Valid scores were transformed as ln(x + 1), with negative missing/refusal codes excluded. Pairs matching used round 1 only. Cognitive Models C1–C3 used the primary Cox covariate sets, without a survival outcome. Coefficients are changes in ln(score+1) per SD of baseline OCN. Outcome-specific complete records were used; neither scores nor covariates were imputed. These concurrent associations cannot establish temporal direction.

### 2.5 Neuroimaging phenotypes

The OCN–MRI associations in Figure 2B and Supplementary Figure S1 used structural MRI measurements from the first imaging assessment (instance 2) in the full OCN cohort of 44,768 participants. No baseline neurological exclusions were applied to this descriptive MRI analysis, which is separate from the incident-dementia cohort. Repeat-imaging measurements from instance 3 were not included in these regressions. We analyzed left, right, and total hippocampal volumes; grey matter, white matter, and total brain volumes; ventricular cerebrospinal fluid (CSF); left and right thalamic and amygdala volumes; and total white matter hyperintensity (WMH) volume. Total hippocampal volume was calculated by summing the left and right measurements before transformation. All MRI outcomes were natural-log transformed; non-positive volumes were treated as missing. The fields used were unnormalized volume measures in mm^3^, not the UK Biobank head-size-normalized global-volume fields. Intracranial volume, the T1 head-size scaling factor, and imaging-center information were unavailable in the MRI analytical dataset and were not included. Individual imaging dates were available only through a partial linkage used for Supplementary Table S2; the blood-sampling-to-MRI interval was not included in these regressions. Baseline age and age-squared were included, but neither imaging age nor the interval was substituted by baseline age. MRI was obtained after the OCN measurement, and scan-to-diagnosis ordering could not be verified throughout the sample. These associations remain exploratory and are not interpreted as causal mediators of incident dementia.

**Figure 2:**
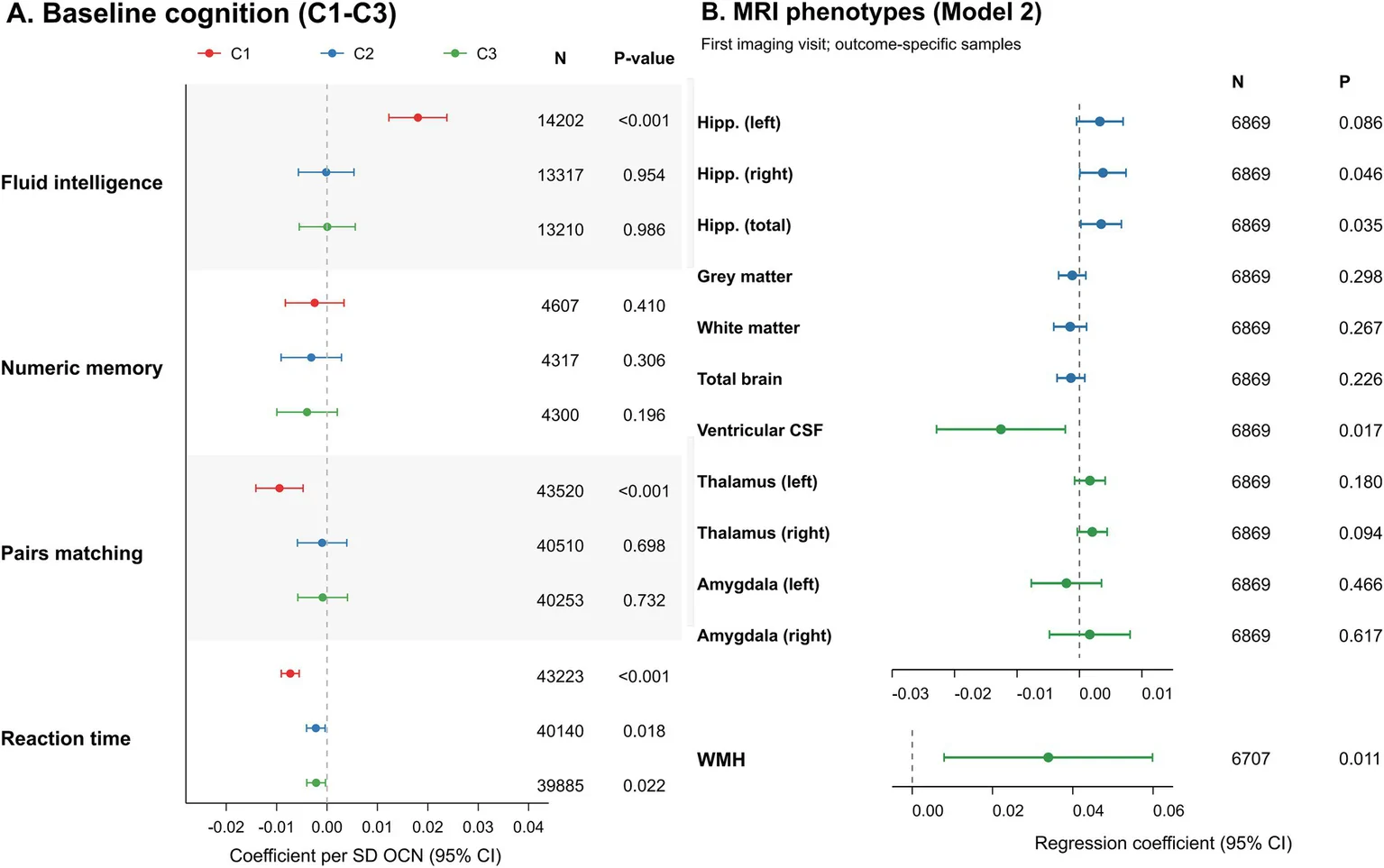
Baseline cognition and later structural MRI associations. **(A)** Cognitive models C1–C3 in the longitudinal cohort. Model colors are red C1, blue C2, and green C3. Cognitive outcomes are ln(*score* + 1); coefficients are per SD of baseline OCN. C1 includes age, age-squared, sex, and BMI; C2 adds education, Townsend, smoking, alcohol, activity, and ethnicity; C3 adds standard AD PRS, phased APOE ε4, and baseline hypertension, diabetes, and stroke. Points and horizontal lines show coefficients and 95% CIs; *N* and nominal *P* are printed. C3 Holm *p* values are 1.000 for fluid intelligence, 0.587 for numeric memory, 1.000 for pairs matching, and 0.090 for reaction time. **(B)** MRI associations use Model 2 and instance-2 measurements in the full OCN cohort. OCN is per NPX unit for ln(*volume*); MRI M2 includes age, age-squared, sex, BMI, qualifications, Townsend, smoking, alcohol, activity, ethnicity, and handedness. *N* = 6,869 for non-WMH volumes and 6,707 for WMH. Nominal *p*-values are displayed; BH *q* across 12 outcomes are in Supplementary Table S3. No head size, imaging center or blood-to-MRI interval was available for adjustment. Matched MRI models are in Supplementary Figure S1 and Supplementary Table S3. The two panels use different OCN scales and populations.

### 2.6 Bone mineral density assessment

Heel bone mineral density (BMD), measured by quantitative ultrasound at instance 0, was the primary skeletal measure. Later-visit DXA measurements were examined separately as exploratory phenotypes. In skeletal and PRS regressions, OCN and each outcome were standardized, and models adjusted for baseline age, sex, BMI, and recruitment center. Assay batch information was unavailable and was not adjusted for. Later DXA associations are not concurrent baseline measurements.

### 2.7 Genetic risk assessment

Genetic-score specifications differed between analysis families. Primary Cox Model 3 used the Standard PRS for Alzheimer’s disease (AD). MRI Model 3 used Standard AD PRS (Field 26,206), Enhanced apolipoprotein A1 PRS (Field 26,208), and Enhanced apolipoprotein B PRS (Field 26,209), entered as continuous scores. Enhanced AD PRS (Field 26,207) was examined in separate exploratory associations and was not included in MRI models. Apolipoprotein A1/B scores are distinct from APOE ε4 carrier status. APOE ε4 status was not established in the MRI model dataset, and these models did not adjust for APOE. The bulk-genotyping indicator (Field 22,418) was not used as an APOE genotype. Exploratory PRS–dementia comparisons are shown in Supplementary Figure S2 and do not define the primary adjustment model.

### 2.8 Covariates

The primary prospective Cox analysis used the following progressive adjustment models. Model 1 was minimally adjusted for basic demographic factors: chronological age, age-squared, sex, and body mass index (BMI). Model 2 further incorporated socioeconomic status and lifestyle factors, including qualifications (education level, utilized as a robust proxy for cognitive reserve), Townsend deprivation index, smoking status, alcohol drinking frequency, and moderate physical activity, alongside ethnic background. Due to the vast majority of participants being of White ancestry and the extremely low number of incident dementia events across other individual ethnic groups, ethnic background was dichotomized as “White versus other” to maintain statistical power and model stability. Model 3 (Fully adjusted model) additionally accounted for genetic predispositions and core cardiometabolic comorbidities: the standard polygenic risk score for AD (PRS-AD), APOE ε4 carrier status, and a history of hypertension (I10), diabetes (E10-E11), and stroke or cerebrovascular disease (I60-I64).

MRI model definitions were specified separately from the primary Cox models. MRI Model 1 adjusted for baseline age, age-squared, sex, and BMI. MRI Model 2 additionally adjusted for qualifications, Townsend deprivation index, smoking status, alcohol drinking frequency, moderate physical activity, ethnic background, and baseline handedness; handedness was included for every MRI outcome. MRI Model 3 additionally included the three PRS variables specified in Section 2.7 and baseline diagnosis indicators for the exact ICD-10 codes listed in the Supplementary Methods. It therefore combined genetic-score and disease-history adjustment. An intermediate Model 2 + PRS added only the three genetic scores. For each outcome, Models 1, 2, 2 + PRS, and 3 were compared in the identical Model 3 complete-case sample. Figure 2B presents Model 2 in its larger outcome-specific complete-case sample; Supplementary Figure S1 and Supplementary Table S3 report the matched-sample comparisons. No clinical, genetic, or MRI values were imputed.

Baseline cognitive associations were estimated using Models C1–C3 in the prospective cohort (Supplementary Table S4). C1 includes age, age-squared, sex and BMI; C2 adds education, Townsend deprivation, smoking, alcohol, activity and White-versus-other ethnicity; C3 adds Standard AD PRS, phased APOE ε4 status and baseline hypertension, diabetes and stroke. These cognitive models differ from MRI Models M1–M3.

Sequentially adjusted Cox sensitivity models used the same prospective cohort, outcome dates and administrative cutoff as the primary analysis: unadjusted, age-adjusted, age-and-sex-adjusted, and age-sex-Standard-AD-PRS-adjusted models (Supplementary Table S5). Subgroup comparisons in Supplementary Tables S6, S7 used different available-case populations and outcome definitions and are interpreted as descriptive comparisons rather than fully adjusted prospective estimates.

Renal function measures, including serum creatinine, cystatin C, and estimated glomerular filtration rate (eGFR), were not available in the analytical extract used for this study and were therefore not included in the adjustment models.

### 2.9 Exploratory multi-omics and statistical path-model screens

Molecular analyses used measured baseline features only. Protein data were taken from the baseline measurement block, with repeat-visit blocks excluded. Metabolites used columns labeled Instance 0. Repeated molecular measurements were not averaged, and later values did not substitute for missing baseline values. We screened 2,922 non-OCN baseline protein columns and 251 metabolites using feature-specific observed pairs. Whole-profile nonmeasurement was distinguished from isolated missing values (Supplementary Table S8). No values were imputed in correlation screens, clustering, or Random Forest analyses.

For observed-only multivariate analyses, baseline features with at least 90% observed values among participants with a measured panel were retained, followed by complete-record selection across those features. This yielded 890 proteins in 14,109 participants and all 251 metabolites in 22,609 participants. There were no participants with every one of the 2,922 protein columns observed; thus, the complete protein matrix is an availability-filtered sensitivity analysis, not a complete-case replication of the entire panel. Scaling, UMAP (30 neighbors, min_dist = 0.3; seed 42; 200 epochs), and K-means (Lloyd algorithm; nstart = 25; maximum 1,000 iterations; seed 123) were fitted to each complete matrix. Both *k* = 2 and *k* = 4 were evaluated. Full-matrix UMAP parameter checks used 15/0.1 and 50/0.5. Twenty participant bootstrap resamples of a fixed 3,000-person evaluation subset fitted scaling, UMAP and clustering within each resample; ARI was evaluated in the unique resampled participants against the subset reference. Availability-filtered feature sets were held fixed. This tests algorithmic stability conditional on the measured sample, not biological reproducibility.

Random Forest regression predicted continuous baseline OCN from the observed complete molecular matrices, using ranger with 300 trees, mtry = floor(sqrt(number of features)), minimum node size 5 and permutation importance. Fivefold cross-validation trained each forest within its training fold; the target was OCN, not dementia. Every baseline feature was also tested using feature-specific observed Spearman correlation and partial rank correlation adjusted for age and sex. Panel-wise Bonferroni correction used 2,922 protein and 251 metabolite tests. WGCNA assignments based on aggregate molecular inputs are reported separately in Table S9; their measurement visits and missing-data processing differ from the baseline screens. Grey denotes nonassignment under the network settings and does not imply a distinct biological process or independence. Molecular correlations were not interpreted as fractions of dementia risk explained.

Baseline molecular correlations were also described separately in participants with a recorded incident dementia event by November 27, 2024 and those without an event by their censoring date in the prospective extract. These are outcome-stratified descriptions, not formal tests of correlation differences or dementia-specific mechanisms. No later molecular measurements or within-person transition were inferred.

Pathway over-representation used baseline proteins that survived age- and sex-adjusted panel-wise Bonferroni correction, intersected with the assay-background gene mapping. Elastic Net features were not added to the pathway foreground. The measured mapped background comprised 2,911 gene symbols (2,872 KEGG-mapped Entrez IDs); the foreground contained 1,011 symbols (1,001 Entrez IDs). One-sided hypergeometric tests were BH-adjusted across 321 eligible KEGG and 1,130 Reactome pathways using the official gene-set releases used for this analysis. Pathways with 5–500 background genes were tested.

Elastic Net was fitted separately in participants of the prospective cohort with measured baseline OCN, heel BMD and all 890 retained baseline protein features (*N* = 7,923; 225 incident dementia events). The positive composite required OCN above the cohort median (NPX > 0), BMD below its cohort median (0.527 g/cm^2^), and incident dementia before censoring; 70 participants met all three conditions. Binomial glmnet used alpha = 0.5, standardized predictors, seed 20,261,007, ten class-stratified folds, AUC selection and lambda.min. This outcome partly incorporates OCN itself, so it is neither an independent dementia classifier nor evidence of causal drivers. Its selected proteins were excluded from pathway enrichment (Supplementary Table S10).

Additionally, we explored BCAA patterns using the independent Mayo Clinic plasma metabolomics cohort ST000046 (*n* = 15 each for Alzheimer’s disease, mild cognitive impairment, and cognitively normal). Log2-transformed LC–MS intensities for leucine, isoleucine, and valine, alongside an equally weighted standardized composite BCAA score, were compared between groups using Welch’s two-sample t-tests with BH correction. This cross-sectional analysis assessed the generalizability of the BCAA disease contrast independently of OCN.

### 2.10 Statistical analysis

Participant characteristics were summarized as means (SD), medians [interquartile ranges], or counts (percentages). Clinical models excluded unknown/refusal categorical codes without imputation or Unknown factor levels. Valid negative Townsend values were included. Qualifications [−7] denotes “None of the above”; unknown/refusal codes were excluded. Primary models used phased APOE data linked by participant ID from the full cohort. SNP-labelled dosage columns and the bulk-genotyping indicator were not used to infer APOE ε4 status. Genetic principal components were unavailable in the analytical dataset and were not included in the models. MRI-specific coding is detailed in Supplementary Methods 2A.

Baseline cognitive associations used C1–C3 linear regressions of ln(score+1) in observed complete records. A negative-binomial sensitivity analysis examined baseline pairs-matching error counts using the same C1–C3 covariates and per-SD OCN scale. Source distribution diagnostics are provided in Supplementary Figure S3. The LCMM analysis used participants with observations at two or more distinct visits, with one value per domain per visit. Fluid intelligence, numeric memory and reaction time were transformed as ln(x + 1) and scaled for fitting. Time was the visit index 0–3, not years, because individual follow-up visit dates were unavailable throughout the extract. Random-intercept, linear-visit hlme models with 1–3 classes were evaluated; two- and three-class fits used ten random starts, a 20-iteration search and up to 200 final iterations. Convergence code 1 was required. BIC, class size, posterior probabilities and entropy were reported. Multiple classes with any class below 5% were not used for subsequent class-membership regression. This is a model-adequacy rule, not proof of a single biological trajectory. Pairs matching was not used for trajectory inference because zero inflation and within-visit repeated rounds do not provide independent follow-up occasions.

Primary Cox models used the baseline covariate hierarchy, per-SD OCN and Efron ties. Schoenfeld tests were reported for every Model 3 term and globally. Because the all-cause model showed departures for age and alcohol and the AD OCN term was non-proportional, sensitivities used flexible age splines, age-band/alcohol-stratified baseline hazards, and separate OCN effects during 0–5 and >5 years. Recruitment-center-stratified Cox models and exclusion of baseline stroke were additionally fitted. Assay batch was unavailable in the extract. Model-specific N, events, precision and primary continuous OCN estimates are consolidated in Supplementary Table S11; diagnostics and sensitivity results are in Supplementary Table S12. Restricted cubic spline displays in Supplementary Figure S4 are descriptive and are not substitutes for the continuous primary estimates.

Incremental discrimination was assessed in the primary Model 3 complete-case sample (*N* = 40,253; 1,095 events) using the prospective outcome dates and administrative cutoff. Fivefold cross-validation compared models with and without OCN in identical training and test participants, using Harrell C with higher linear predictors denoting higher risk. Mean fold-specific C and the paired change are reported in Supplementary Table S13. An exploratory bidirectional stepwise Cox model included heel BMD, used AIC, and had *N* = 22,882 with 679 events (Supplementary Table S14); no Unknown smoking or alcohol categories were fitted.

All-cause dementia was the single primary survival outcome. Holm correction was applied to the three Model 3 secondary outcomes (AD, VaD and FTD), and separately to four C3 cognitive tests. For MRI, BH correction was reported across 12 outcomes within each displayed model/sample family, while nominal *p-*values were retained. Molecular screens used panel-wise Bonferroni correction and pathways used BH correction. Other model stages, subgroup, interaction, clustering, feature-selection and sensitivity analyses remain exploratory; correction within one family does not provide study-wide error control.

We fitted exploratory statistical path models using only initial-assessment (instance 0) measurements in the cohort. Heel BMD was taken from the numerical Heel bone mineral density field, without substitution by a *T*-score or a later-visit measure. OCN was taken from baseline Olink BGLAP. Cognitive outcomes were baseline reaction time and the number of incorrect matches in pairs-matching round 1. Fluid intelligence and numeric memory were not analyzed in these path models because neither had overlapping observations with the selected instance-0 Heel BMD field in the analytical dataset. Cognitive outcomes were transformed as log(x + 1), and OCN, BMD, and transformed cognition were standardized within each outcome-specific complete-case sample. Models adjusted for age, sex, BMI, Townsend deprivation index, and university-degree status. The same participants were used for the two alternative specifications within each cognitive outcome. These limited-adjustment, exploratory models do not establish temporal direction or causal mediation.

Two- and five-year landmark analyses retained the primary Model 3 specification and November 27, 2024 cutoff; follow-up restarted at each landmark in alive, uncensored, dementia-free participants. Primary cognition-adjusted checks are shown in Supplementary Table S15. The White-restricted Cox sensitivity was fitted with the same clinical records, baseline exclusions, covariates and censoring date as the primary model; White-versus-other was removed because ethnicity was constant. The analysis included 37,828 complete records and 1,063 events. Competing-risk comparisons use a separate minimally adjusted specification and are interpreted descriptively. Analyses used R 4.6.1 and Python. Code and aggregate outputs are provided with the Supplementary materials.

## 3 Results

### 3.1 Baseline characteristics

The OCN cohort comprised 44,768 participants (Figure 1). The longitudinal cohort contained 43,942 participants after the study exclusions; model-specific analyses used observed complete records. Incident all-cause dementia ascertainment identified 1,266 events before model-specific complete-record selection, with follow-up censored at diagnosis, death, or November 27, 2024. Tables 1, 2 describe participants by baseline OCN quartile and incident all-cause dementia, respectively. Clinical histories in these tables use first report dates on or before baseline; later imaging measurements are identified separately. Field definitions and coding are in Supplementary Table S1, S16, and molecular availability is described in Table S8. Cohort and recorded-subtype comparisons are in Supplementary Tables S17, S18.

**Table 1:** Participant characteristics by baseline plasma OCN quartile.

Characteristic	Q1 *N* = 11,192	Q2 *N* = 11,192	Q3 *N* = 11,192	Q4 *N* = 11,192	*p-*value
Baseline demographic and lifestyle characteristics					
Relative plasma OCN, NPX; median [IQR]	−1.07 [−1.49, −0.81]	−0.27 [−0.42, −0.12]	0.27 [0.13, 0.42]	1.00 [0.77, 1.35]	<0.001
Age, years; mean (SD)	56.32 (8.50)	56.59 (8.28)	56.68 (8.18)	57.73 (7.72)	<0.001
BMI, kg/m^2^; mean (SD)	27.05 (4.72)	27.48 (4.72)	27.67 (4.81)	27.66 (4.97)	<0.001
Townsend index; median [IQR]	−1.69 [−3.51, 1.38]	−2.13 [−3.63, 0.62]	−2.18 [−3.68, 0.53]	−2.09 [−3.58, 0.63]	<0.001
Sex, *n* (%)					<0.001
Female	5,574 (49.8)	5,365 (47.9)	6,037 (53.9)	7,230 (64.6)	
Male	5,618 (50.2)	5,827 (52.1)	5,155 (46.1)	3,962 (35.4)	
Qualifications codes 1/2/5/6, *n* (%)	7,531 (68.6)	7,359 (66.9)	7,159 (65.0)	6,638 (60.2)	<0.001
Moderate activity, days/week; mean (SD)	3.69 (2.36)	3.65 (2.33)	3.62 (2.32)	3.57 (2.37)	0.004
Former or current smoker, *n* (%)	5,531 (49.7)	5,245 (47.1)	4,940 (44.3)	4,698 (42.2)	<0.001
Handedness, *n* (%)					0.283
Right-handed	9,917 (88.8)	9,912 (88.7)	9,917 (88.7)	10,022 (89.6)	
Left-handed	1,060 (9.5)	1,071 (9.6)	1,078 (9.6)	977 (8.7)	
Uses both hands equally	191 (1.7)	187 (1.7)	186 (1.7)	181 (1.6)	
Prefer not to answer, *n* (% group)	3 (0.0)	10 (0.1)	4 (0.0)	4 (0.0)	
Handedness not recorded, *n* (% group)	21 (0.2)	12 (0.1)	7 (0.1)	8 (0.1)	
Alcohol frequency, n (%)					<0.001
Never	1,145 (10.3)	842 (7.5)	826 (7.4)	1,079 (9.7)	
Special occasions only	1,276 (11.4)	1,156 (10.4)	1,270 (11.4)	1,587 (14.2)	
One to three times/month	1,116 (10.0)	1,120 (10.0)	1,228 (11.0)	1,374 (12.3)	
Once or twice/week	2,681 (24.0)	2,907 (26.0)	3,056 (27.4)	2,957 (26.5)	
Three or four times/week	2,535 (22.7)	2,666 (23.9)	2,571 (23.0)	2,230 (20.0)	
Daily or almost daily	2,405 (21.6)	2,476 (22.2)	2,221 (19.9)	1,948 (17.4)	
Ethnic background, *n* (%)					<0.001
White	9,694 (87.2)	10,505 (94.3)	10,685 (95.8)	10,737 (96.4)	
Black	683 (6.1)	151 (1.4)	120 (1.1)	138 (1.2)	
Asian (including Chinese)	390 (3.5)	277 (2.5)	189 (1.7)	138 (1.2)	
Mixed ethnicity	106 (1.0)	72 (0.6)	58 (0.5)	55 (0.5)	
Other ethnicity	244 (2.2)	131 (1.2)	102 (0.9)	75 (0.7)	
Ethnicity not reported, *n* (% group)	75 (0.7)	56 (0.5)	38 (0.3)	49 (0.4)	
Baseline APOE and polygenic risk scores					
Any phased APOE ε4 carrier, n (%)	2,781 (25.0)	2,732 (24.6)	2,770 (24.9)	2,857 (25.7)	0.260
APOE genotype group, n (%)					0.082
Non-carrier	8,325 (75.0)	8,391 (75.4)	8,343 (75.1)	8,259 (74.3)	
E2/E4 or E3/E4	2,481 (22.3)	2,423 (21.8)	2,488 (22.4)	2,593 (23.3)	
E4/E4	300 (2.7)	309 (2.8)	282 (2.5)	264 (2.4)	
Standard AD PRS; mean (SD)	0.04 (1.02)	0.07 (1.01)	0.07 (1.02)	0.09 (1.01)	0.029
Enhanced AD PRS; mean (SD)	0.01 (1.02)	0.05 (1.03)	0.05 (1.02)	0.04 (1.02)	0.495
Enhanced apolipoprotein A1 PRS; mean (SD)	0.01 (1.05)	0.00 (1.04)	0.01 (1.04)	0.01 (1.07)	0.991
Enhanced apolipoprotein B PRS; mean (SD)	−0.06 (1.08)	−0.05 (1.05)	−0.01 (1.06)	−0.02 (1.05)	0.172
Clinical histories recorded on or before baseline					
Diabetes (E10–E14), *n* (%)	1,061 (9.5)	602 (5.4)	440 (3.9)	401 (3.6)	<0.001
Diabetes in primary model (E10/E11), *n* (%)	602 (5.4)	337 (3.0)	260 (2.3)	273 (2.4)	<0.001
Hypertension (I10/I15), *n* (%)	3,211 (28.7)	3,033 (27.1)	3,047 (27.2)	3,263 (29.2)	<0.001
Hypertension in primary model (I10), *n* (%)	3,211 (28.7)	3,033 (27.1)	3,047 (27.2)	3,263 (29.2)	<0.001
Cerebrovascular disorders, *n* (%)	278 (2.5)	238 (2.1)	245 (2.2)	365 (3.3)	<0.001
Stroke in primary model (I60–I64), *n* (%)	197 (1.8)	172 (1.5)	174 (1.6)	259 (2.3)	<0.001
Demyelinating disorders, *n* (%)	100 (0.9)	91 (0.8)	86 (0.8)	89 (0.8)	0.753
Neurodegenerative disorders, *n* (%)	110 (1.0)	101 (0.9)	107 (1.0)	122 (1.1)	0.542
Organic brain disorders, *n* (%)	100 (0.9)	96 (0.9)	102 (0.9)	139 (1.2)	0.011
Mental disorders, *n* (%)	2,366 (21.1)	2,145 (19.2)	2,125 (19.0)	2,287 (20.4)	<0.001
Cognition at the initial assessment					
Fluid intelligence, raw score; median [IQR]	5.00 [4.00, 7.00]	5.00 [4.00, 7.00]	5.00 [4.00, 7.00]	5.00 [4.00, 7.00]	0.002
Fluid intelligence, ln(score+1); mean (SD)	1.77 (0.37)	1.80 (0.35)	1.80 (0.34)	1.79 (0.35)	0.002
Numeric memory, raw score; median [IQR]	7.00 [6.00, 8.00]	7.00 [6.00, 7.25]	7.00 [6.00, 7.00]	7.00 [6.00, 7.00]	0.006
Numeric memory, ln(score+1); mean (SD)	2.02 (0.20)	2.02 (0.19)	2.02 (0.19)	2.01 (0.19)	0.006
Reaction time, raw ms; median [IQR]	543.00 [484.00, 621.00]	536.00 [480.00, 613.00]	539.00 [480.00, 613.00]	547.00 [488.00, 622.00]	<0.001
Reaction time, ln(score+1); mean (SD)	6.32 (0.20)	6.31 (0.19)	6.31 (0.20)	6.33 (0.19)	<0.001
Pairs matching, round 1, raw errors; median [IQR]	0.00 [0.00, 1.00]	0.00 [0.00, 1.00]	0.00 [0.00, 1.00]	0.00 [0.00, 1.00]	<0.001
Pairs matching, round 1, ln(score+1); mean (SD)	0.29 (0.51)	0.28 (0.49)	0.28 (0.49)	0.30 (0.50)	<0.001
Pairs matching, zero errors, *n* (%)	7,904 (71.1)	8,052 (72.3)	8,010 (71.9)	7,791 (69.9)	<0.001
Skeletal measurements at the initial assessment					
Heel BMD, g/cm^2^; mean (SD)	0.56 (0.15)	0.55 (0.14)	0.54 (0.14)	0.52 (0.14)	<0.001
Heel BMD, g/cm^2^; median [IQR]	0.54 [0.46, 0.63]	0.54 [0.46, 0.62]	0.53 [0.45, 0.61]	0.50 [0.43, 0.59]	<0.001
Heel BMD T-score; mean (SD)	−0.21 (1.33)	−0.25 (1.24)	−0.36 (1.25)	−0.58 (1.23)	<0.001
Structural MRI at the later first imaging visit					
Left hippocampus, mm^3^; mean (SD)	3,693 (501)	3,693 (499)	3,720 (497)	3,679 (495)	0.108
Right hippocampus, mm^3^; mean (SD)	3,799 (514)	3,804 (510)	3,837 (518)	3,795 (512)	0.234
Total hippocampus, mm^3^; mean (SD)	7,492 (929)	7,497 (910)	7,557 (929)	7,474 (923)	0.111
Left thalamus, mm^3^; mean (SD)	7,605 (786)	7,637 (764)	7,655 (784)	7,542 (759)	<0.001
Right thalamus, mm^3^; mean (SD)	7,420 (757)	7,453 (742)	7,462 (762)	7,362 (734)	<0.001
Left amygdala, mm^3^; mean (SD)	1,251 (250)	1,262 (253)	1,258 (246)	1,227 (243)	<0.001
Right amygdala, mm^3^; mean (SD)	1,214 (274)	1,217 (272)	1,222 (264)	1,190 (267)	<0.001
WMH, mm^3^; mean (SD)	5,960 (8,516)	6,116 (8,507)	6,053 (8,654)	6,468 (8,349)	0.003
Ventricular CSF, mm^3^; mean (SD)	40,529 (19,301)	40,200 (18,627)	38,957 (17,858)	38,018 (17,312)	<0.001
Grey matter, mm^3^; mean (SD)	604,364 (58,843)	608,066 (56,253)	608,277 (59,189)	595,082 (58,083)	<0.001
White matter, mm^3^; mean (SD)	541,054 (64,121)	543,125 (60,687)	542,010 (63,590)	529,253 (61,586)	<0.001
Total brain, mm^3^; mean (SD)	1,145,418 (115,563)	1,151,191 (109,572)	1,150,287 (116,689)	1,124,335 (113,388)	<0.001
Recorded dementia diagnoses in full clinical records (all report dates)					
All-cause dementia, *n* (%)	323 (2.9)	334 (3.0)	310 (2.8)	411 (3.7)	<0.001
AD, *n* (%)	158 (1.4)	171 (1.5)	158 (1.4)	223 (2.0)	<0.001
VaD, *n* (%)	63 (0.6)	71 (0.6)	58 (0.5)	87 (0.8)	0.073
FTD, *n* (%)	49 (0.4)	57 (0.5)	50 (0.4)	52 (0.5)	0.87

Values are mean (SD), median [25th, 75th percentiles], or n (%). Categorical percentages use observed eligible measurements. Refusal/nonresponse rows use the full group denominator. Comparisons are descriptive, unadjusted Kruskal–Wallis tests for continuous measurements and Pearson chi-square tests for categorical variables; category-level rows share their parent variable’s test. A dash indicates no estimable comparison. No study-wide correction is applied to these descriptive tests. Clinical histories use reports on or before baseline. Diabetes, hypertension, and cerebrovascular disorders are shown both as broader diagnostic groups and using the primary Cox definitions (E10/E11, I10, and I60–I64, respectively). The full diagnostic code groups are specified in Supplementary Methods 2C. OCN, cognition, heel BMD, and automated heel BMD T-score are initial-assessment measurements (instance 0). Negative cognitive missing/refusal codes and nonpositive BMD values are excluded; valid negative Townsend, PRS, and T-score values are retained. No missing value is filled. APOE categories use the phased cohort genotype; apolipoprotein A1/B PRS are distinct from APOE. MRI volumes are from the later first imaging assessment (instance 2), expressed in mm^3^; they are not baseline measurements. Total hippocampal volume is the sum of the observed left and right volumes. Valid measurements are summarized descriptively, without applying MRI regression covariate exclusions. The full OCN cohort comprises 44,768 participants, with 11,192 in each OCN quartile. Recorded-dementia summaries cover all available report dates and include baseline cases; they are distinct from the incident events used in the primary Cox models. AD, Alzheimer’s disease; BMD, bone mineral density; BMI, body mass index; CSF, cerebrospinal fluid; FTD, frontotemporal dementia; IQR, interquartile range; MRI, magnetic resonance imaging; NPX, Normalized Protein eXpression; OCN, osteocalcin; PRS, polygenic risk score; SD, standard deviation; VaD, vascular dementia; WMH, white matter hyperintensities.

**Table 2:** Participant characteristics by incident all-cause dementia in the longitudinal cohort.

Characteristic	No incident dementia *N* = 42,675	Incident dementia *N* = 1,266	*P-*value
Baseline demographic and lifestyle characteristics			
Relative plasma OCN, NPX; median [IQR]	0.00 [−0.60, 0.58]	0.04 [−0.56, 0.70]	0.007
Age, years; mean (SD)	56.57 (8.16)	64.57 (5.11)	<0.001
BMI, kg/m^2^; mean (SD)	27.46 (4.81)	27.56 (4.75)	0.183
Townsend index; median [IQR]	−2.03 [−3.60, 0.78]	−2.08 [−3.64, 1.07]	0.919
Sex, n (%)			0.141
Female	23,073 (54.1)	658 (52.0)	
Male	19,602 (45.9)	608 (48.0)	
Qualifications codes 1/2/5/6, *n* (%)	27,559 (65.7)	616 (50.3)	<0.001
Moderate activity, days/week; mean (SD)	3.64 (2.34)	3.93 (2.41)	<0.001
Former or current smoker, *n* (%)	19,362 (45.6)	655 (51.9)	<0.001
Handedness, *n* (%)			0.579
Right-handed	37,909 (89.0)	1,132 (89.4)	
Left-handed	3,996 (9.4)	110 (8.7)	
Uses both hands equally	704 (1.7)	24 (1.9)	
Prefer not to answer, *n* (% group)	20 (0.0)	0 (0.0)	
Handedness not recorded, *n* (% group)	46 (0.1)	0 (0.0)	
Alcohol frequency, *n* (%)			<0.001
Never	3,624 (8.5)	168 (13.3)	
Special occasions only	4,969 (11.7)	196 (15.5)	
One to three times/month	4,601 (10.8)	133 (10.5)	
Once or twice/week	11,119 (26.1)	281 (22.2)	
Three or four times/week	9,622 (22.6)	238 (18.8)	
Daily or almost daily	8,650 (20.3)	247 (19.6)	
Ethnic background, *n* (%)			<0.001
White	39,599 (93.3)	1,219 (96.5)	
Black	1,064 (2.5)	23 (1.8)	
Asian (including Chinese)	978 (2.3)	6 (0.5)	
Mixed ethnicity	282 (0.7)	5 (0.4)	
Other ethnicity	540 (1.3)	10 (0.8)	
Ethnicity not reported, *n* (% group)	212 (0.5)	3 (0.2)	
Baseline APOE and polygenic risk scores			
Any phased APOE ε4 carrier, *n* (%)	10,309 (24.3)	644 (51.4)	<0.001
APOE genotype group, *n* (%)			<0.001
Non-carrier	32,070 (75.7)	610 (48.6)	
E2/E4 or E3/E4	9,378 (22.1)	442 (35.2)	
E4/E4	931 (2.2)	202 (16.1)	
Standard AD PRS; mean (SD)	0.04 (1.00)	0.85 (1.26)	<0.001
Enhanced AD PRS; mean (SD)	0.02 (1.01)	0.74 (1.29)	<0.001
Enhanced apolipoprotein A1 PRS; mean (SD)	0.01 (1.05)	0.01 (1.06)	0.859
Enhanced apolipoprotein B PRS; mean (SD)	−0.04 (1.06)	0.24 (1.06)	<0.001
Clinical histories recorded on or before baseline			
Diabetes (E10–E14), n (%)	2,257 (5.3)	153 (12.1)	<0.001
Diabetes in primary model (E10/E11), n (%)	1,188 (2.8)	104 (8.2)	<0.001
Hypertension (I10/I15), n (%)	11,746 (27.5)	544 (43.0)	<0.001
Hypertension in primary model (I10), n (%)	11,746 (27.5)	544 (43.0)	<0.001
Cerebrovascular disorders, n (%)	1,008 (2.4)	68 (5.4)	<0.001
Stroke in primary model (I60–I64), n (%)	711 (1.7)	48 (3.8)	<0.001
Demyelinating disorders, n (%)	0 (0.0)	0 (0.0)	—
Neurodegenerative disorders, n (%)	0 (0.0)	0 (0.0)	—
Organic brain disorders, n (%)	379 (0.9)	15 (1.2)	0.270
Mental disorders, n (%)	5,944 (13.9)	211 (16.7)	0.006
Cognition at the initial assessment			
Fluid intelligence, raw score; median [IQR]	5.00 [4.00, 7.00]	4.00 [3.00, 6.00]	<0.001
Fluid intelligence, ln(score+1); mean (SD)	1.79 (0.35)	1.64 (0.39)	<0.001
Numeric memory, raw score; median [IQR]	7.00 [6.00, 8.00]	6.00 [5.00, 7.00]	<0.001
Numeric memory, ln(score+1); mean (SD)	2.02 (0.19)	1.94 (0.25)	<0.001
Reaction time, raw ms; median [IQR]	539.00 [481.00, 614.00]	594.00 [525.50, 687.00]	<0.001
Reaction time, ln(score+1); mean (SD)	6.31 (0.19)	6.41 (0.23)	<0.001
Pairs matching, round 1, raw errors; median [IQR]	0.00 [0.00, 1.00]	0.00 [0.00, 1.00]	<0.001
Pairs matching, round 1, ln(score+1); mean (SD)	0.28 (0.49)	0.43 (0.58)	<0.001
Pairs matching, zero errors, n (%)	30,432 (71.7)	758 (60.3)	<0.001
Skeletal measurements at the initial assessment			
Heel BMD, g/cm^2^; mean (SD)	0.54 (0.14)	0.52 (0.16)	<0.001
Heel BMD, g/cm^2^; median [IQR]	0.53 [0.45, 0.62]	0.50 [0.42, 0.59]	<0.001
Heel BMD T-score; mean (SD)	−0.34 (1.27)	−0.55 (1.38)	<0.001
Structural MRI at the later first imaging visit			
Left hippocampus, mm^3^; mean (SD)	3,702 (497)	3,373 (588)	0.011
Right hippocampus, mm^3^; mean (SD)	3,815 (510)	3,455 (561)	0.005
Total hippocampus, mm^3^; mean (SD)	7,517 (917)	6,828 (1,091)	0.004
Left thalamus, mm^3^; mean (SD)	7,621 (770)	7,301 (1,027)	0.037
Right thalamus, mm^3^; mean (SD)	7,435 (745)	7,113 (891)	0.057
Left amygdala, mm^3^; mean (SD)	1,252 (248)	1,332 (399)	0.635
Right amygdala, mm^3^; mean (SD)	1,213 (269)	1,267 (406)	0.909
WMH, mm^3^; mean (SD)	5,987 (8,364)	16,251 (18,626)	0.003
Ventricular CSF, mm^3^; mean (SD)	39,317 (18,277)	59,322 (25,943)	<0.001
Grey matter, mm^3^; mean (SD)	604,494 (58,239)	591,708 (75,478)	0.485
White matter, mm^3^; mean (SD)	539,385 (62,615)	549,720 (84,050)	0.447
Total brain, mm^3^; mean (SD)	1,143,879 (114,104)	1,141,429 (153,484)	0.977

Values are mean (SD), median [25th, 75th percentiles], or n (%). Categorical percentages use observed eligible measurements. Refusal/nonresponse rows use the full group denominator. Comparisons are descriptive, unadjusted Kruskal–Wallis tests for continuous measurements and Pearson chi-square tests for categorical variables; category-level rows share their parent variable’s test. A dash indicates no estimable comparison. No study-wide correction is applied to these descriptive tests. Clinical histories use reports on or before baseline. Diabetes, hypertension, and cerebrovascular disorders are shown both as broader diagnostic groups and using the primary Cox definitions (E10/E11, I10, and I60–I64, respectively). The full diagnostic code groups are specified in Supplementary Methods 2C. OCN, cognition, heel BMD, and automated heel BMD T-score are initial-assessment measurements (instance 0). Negative cognitive missing/refusal codes and nonpositive BMD values are excluded; valid negative Townsend, PRS, and T-score values are retained. No missing value is filled. APOE categories use the phased cohort genotype; apolipoprotein A1/B PRS are distinct from APOE. MRI volumes are from the later first imaging assessment (instance 2), expressed in mm^3^; they are not baseline measurements. Total hippocampal volume is the sum of the observed left and right volumes. Valid measurements are summarized descriptively, without applying MRI regression covariate exclusions. The longitudinal cohort comprises 43,942 participants. The comparison uses observed records and the primary baseline exclusions, with follow-up censored at dementia diagnosis, death, or November 27, 2024; 1,266 incident all-cause events were recorded. AD, Alzheimer’s disease; BMD, bone mineral density; BMI, body mass index; CSF, cerebrospinal fluid; FTD, frontotemporal dementia; IQR, interquartile range; MRI, magnetic resonance imaging; NPX, Normalized Protein eXpression; OCN, osteocalcin; PRS, polygenic risk score; SD, standard deviation; VaD, vascular dementia; WMH, white matter hyperintensities.

Participant characteristics by OCN quartile are shown in Table 1. Each quartile includes 11,192 participants. Higher OCN quartiles contained older participants and a larger proportion of women. Baseline diabetes defined by E10/E11 was present in 5.4% of Q1 and 2.4% of Q4 participants. Clinical histories were ascertained from reports on or before the initial assessment. These descriptive comparisons do not establish causal effects of OCN.

Over the follow-up period, 1,266 participants in the longitudinal cohort developed incident all-cause dementia. Unadjusted baseline comparisons showed that incident dementia cases had higher median OCN levels than dementia-free controls (0.04 vs. 0.00 NPX, *p* = 0.007). Incident dementia cases demonstrated lower baseline cognitive performance across domains including fluid intelligence, numeric memory, reaction time, and pairs matching (all *p* < 0.001) (Table 2). Relative OCN comparisons across worst-quartile cognitive groupings are shown in Supplementary Figure S5. The Enhanced Polygenic Risk Score for AD (PRS-AD) was associated with incident AD, whereas associations for lipid-related genetic scores (e.g., PRS-APOEA) were less robust or non-significant (Supplementary Figure S2). Analysis of the neuroimaging subcohort (Supplementary Table S19A) showed that participants with MRI data (*N* = 7,151) were younger, had a higher proportion of advanced education (80% vs. 61%), possessed fewer cardiometabolic comorbidities, and had a lower baseline prevalence of all-cause dementia (0.3% vs. 3.6%, *p* < 0.001) compared to the non-MRI group (*N* = 37,617).

Similarly, baseline comparisons between participants with and without BMD data (Supplementary Table S19B) revealed inherent sub-cohort disparities. Participants evaluated for Heel BMD (*N* = 25,155) exhibited a lower Townsend Deprivation Index (indicating higher socioeconomic status), a significantly higher proportion of White individuals (97% vs. 92%, *p* < 0.001), and slightly lower diabetes prevalence (11% vs. 12%, *p* = 0.020) compared to those without BMD measurements (*N* = 19,613). Furthermore, the BMD sub-cohort showed slightly higher proportions with recorded all-cause dementia (3.3% vs. 2.8%, *p* < 0.001) and Alzheimer’s disease (1.7% vs. 1.4%, *p* = 0.006). Clinical diagnosis entries in these subgroup descriptions cover all available report dates; the primary model histories and main tables use reports on or before baseline.

### 3.2 Associations of baseline OCN with cognitive and imaging phenotypes

The baseline cognitive analysis is Figure 2A and Supplementary Table S4, using baseline instance-0 measures in the prospective extract. In C3, the OCN coefficients were 0.00005 for fluid intelligence (95% CI, −0.00551 to 0.00560; *N* = 13,210; *p* = 0.986), −0.00396 for numeric memory (−0.00996 to 0.00204; *N* = 4,300; *p* = 0.196), −0.00216 for reaction time (−0.00401 to −0.00031; *N* = 39,885; *p* = 0.022), and −0.00086 for pairs matching (−0.00580 to 0.00407; *N* = 40,253; *p* = 0.732), per SD of baseline OCN for ln(score+1). The reaction-time association was small and did not survive Holm correction across four domains (adjusted *p* = 0.090); no C3 cognitive test survived correction. Performance-group descriptions in Figure S5 are separate descriptive comparisons. Cognitive-subset characteristics are described in Supplementary Table S20; model-specific samples are reported in Supplementary Table S4.

The fluid-intelligence coefficient changed from 0.01804 in C1 (*p* < 0.001) to −0.00016 in C2 (*p* = 0.954) and 0.00005 in C3 (*p* = 0.986). The positive minimally adjusted association was therefore not evident after additional socioeconomic and lifestyle adjustment or in the fully adjusted model (Supplementary Figure S6).

For pairs matching, the C3 negative-binomial error-rate ratio was 0.988 (95% CI, 0.967–1.009; *p* = 0.262; Supplementary Table S4B). The count-model check also provided no clear fully adjusted association. Differences across available-case model stages may reflect changes in participant composition as well as covariate adjustment.

In MRI Model 2, higher baseline OCN had a nominal positive association with later total hippocampal volume (*β* = 0.0035; 95% CI, 0.0002–0.0067; *N* = 6,869; *p* = 0.035) and WMH volume (*β* = 0.0339; 95% CI, 0.0079–0.0599; *N* = 6,707; *p* = 0.011) (Figure 2B and Supplementary Table S3A). These coefficients are per one-unit increase in OCN NPX for ln(volume). Participant characteristics of the imaging subset are described in Supplementary Table S21; the model-specific participant counts are provided in Supplementary Table S3C.

In the same 1,969 participants with complete data for total hippocampal volume and all MRI Model 3 covariates, the OCN coefficients were 0.0037 (95% CI, −0.0017 to 0.0092; *p* = 0.179) in Model 1, 0.0002 (−0.0055 to 0.0059; *p* = 0.949) in Model 2, and −0.0004 (−0.0061 to 0.0054; *p* = 0.902) in Model 3. Adding only the PRS variables to Model 2 yielded β = 0.00003 (*p* = 0.992). In the same 1,926 participants with WMH data, the coefficients were 0.0349 (−0.0100 to 0.0799; *p* = 0.128), 0.0329 (−0.0150 to 0.0808; *p* = 0.179), and 0.0292 (−0.0185 to 0.0768; *p* = 0.230), respectively; Model 2 + PRS yielded β = 0.0302 (*p* = 0.216) (Supplementary Figure S1 and Supplementary Table S3B). Thus, the hippocampal Model 2 estimate was already close to zero before adding PRS in the matched subset, and the WMH estimates changed modestly with genetic-score adjustment while remaining imprecise. The difference between the larger and matched samples cannot be attributed specifically to genetic adjustment, and these comparisons do not establish a genetic mechanism.

### 3.3 Longitudinal cognitive trajectories and incident dementia

The repeated-visit analysis included 4,438 participants for fluid intelligence, 744 for numeric memory, and 9,005 for reaction time. All candidate models converged, but the smallest classes in the three-class solutions comprised 0.29, 2.02, and 2.57% of participants, respectively. BIC, candidate class sizes, and posterior assignment summaries are reported in Supplementary Table S22. No multiple-class solution met the requirement that every class include at least 5% of participants; class-membership regressions were therefore not performed. Unequal visit intervals and small latent classes limit interpretation of cognitive trajectories.

Primary continuous OCN estimates are reported in Supplementary Table S11; Figure 3 shows survival curves and quartile Cox estimates in the Model 3 complete-record samples. OCN was not clearly associated with all-cause dementia in Model 3 (HR per SD, 1.052; 95% CI, 0.988–1.120; *p* = 0.117; *N* = 40,253; 1,095 events). AD had HR 1.089 (0.998–1.187; *p* = 0.055), VaD had HR 1.159 (1.010–1.328; *p* = 0.035), and FTD had HR 0.948 (0.797–1.128; *p* = 0.548). Holm-adjusted *p* values for the three secondary outcomes were 0.111, 0.105 and 0.548, respectively. The subtype observations are exploratory.

**Figure 3:**
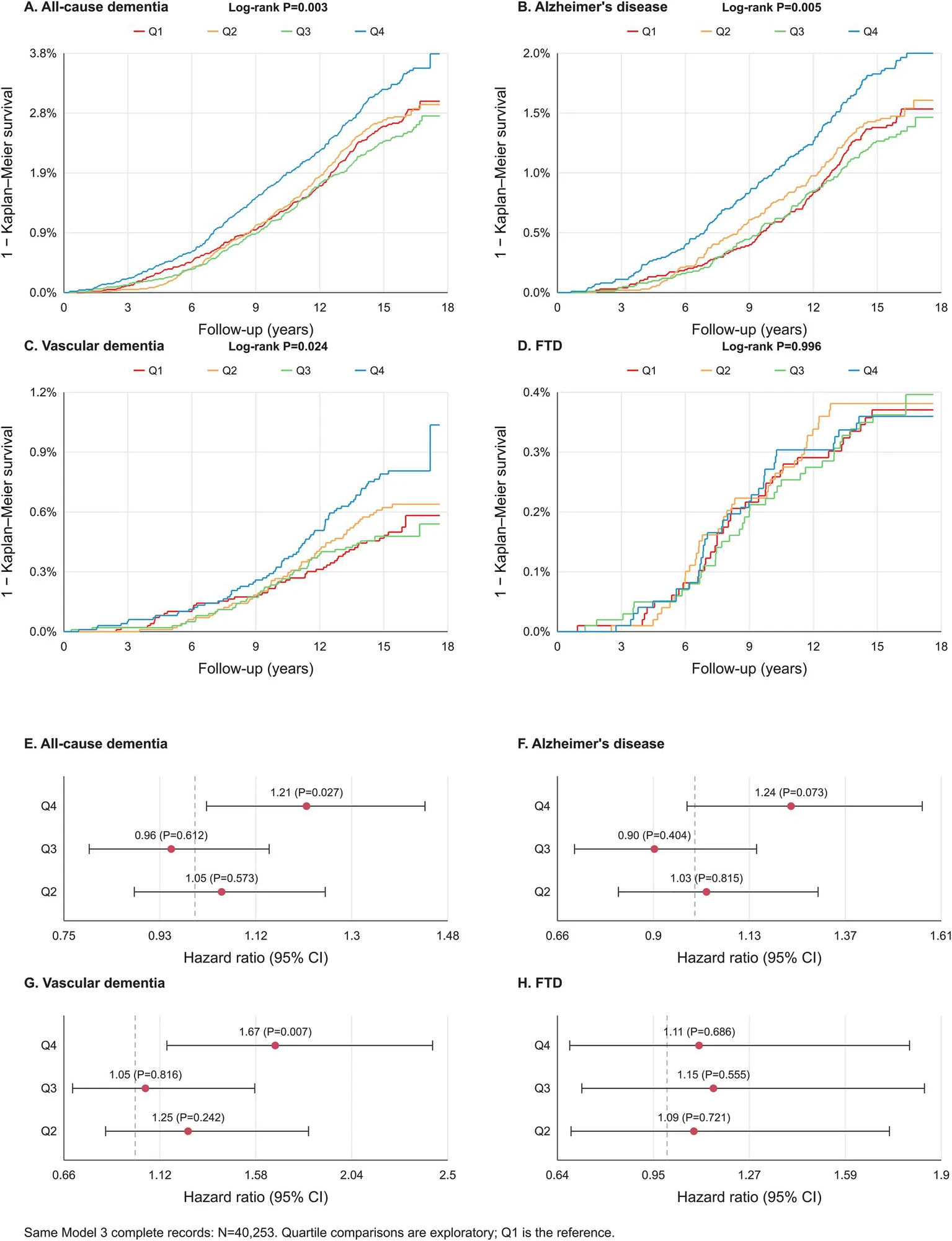
Recorded dementia by baseline OCN quartile in the primary Model 3 complete-record sample. **(A–D)** One minus Kaplan–Meier survival for all-cause dementia, AD, VaD, and FTD. Death is censored in these curves; these are not competing-risk cumulative incidence estimates. Log-rank *p*-values are unadjusted comparisons. **(E–H)** Quartile Cox estimates with the primary Model 3 covariates, with Q1 as reference. Every panel uses 40,253 complete records and the November 27, 2024 cutoff. Points and horizontal lines denote HRs and 95% CIs. Quartile comparisons are exploratory; the primary continuous per-SD OCN estimates and secondary-outcome correction are in Supplementary Table S11. AD temporal variation, PH tests, and stroke/center sensitivities are in Supplementary Table S12. Quartile estimates and log-rank results are additionally reported in Supplementary Table S11.

In the Model 3 quartile analysis, Q4 versus Q1 had HR 1.211 (95% CI, 1.022–1.436; *p* = 0.027) for all-cause dementia and HR 1.674 (1.153–2.430; *p* = 0.007) for VaD. The AD contrast was HR 1.237 (0.981–1.560; *p* = 0.073), and FTD had HR 1.106 (0.679–1.800; *p* = 0.686). These are nominal categorical contrasts among 12 quartile tests, without an additional quartile-family correction. They remain exploratory and do not replace the primary continuous per-SD OCN test (Figure 3 and Supplementary Table S11).

Model 3 had 52.1 events per fitted parameter for all-cause dementia and 27.8 for AD, compared with 11.0 for VaD and 6.8 for FTD (Table S11B). Precision is particularly limited for the latter outcomes; these retrospective precision summaries are not evidence of prospective statistical power.

For all-cause dementia, the OCN Schoenfeld test had *p* = 0.136, but the global test had *p* = 0.013, with age (*p* = 0.012) and alcohol (*p* = 0.046) showing departures. Stratifying baseline hazards jointly by 5-year age bands and alcohol categories gave HR 1.047 (95% CI, 0.984–1.115; *p* = 0.148). For AD, the OCN Schoenfeld test had *p* = 0.014. Its OCN HR was 1.396 (1.087–1.793) during 0–5 years and 1.055 (0.962–1.156) thereafter; the early–late difference test had *p* = 0.038. A single AD HR therefore obscures temporal variation. VaD and FTD OCN Schoenfeld *p-*values were 0.956 and 0.782, respectively (Supplementary Table S12).

The observed-complete-record bidirectional AIC analysis did not retain OCN (*N* = 22,882; 679 events; Supplementary Table S14). Unknown smoking or alcohol factor levels were not fitted. This data-driven model is exploratory and does not establish a causal adjustment set or validate predictive performance.

Age and skeletal-feature associations in Supplementary Table S23 provide descriptive context and are not a mechanistic explanation of variable selection. Centre-adjusted skeletal and genetic associations are reported in Supplementary Table S24. OCN was evaluated continuously; absence of selection in the AIC model does not establish that a biological effect is absent.

Sequential Cox sensitivity models used the same longitudinal cohort and administrative cutoff as the primary analysis (Supplementary Table S5). The cohort contained 43,942 participants and 1,266 recorded events before model-specific exclusions; Model 3 included 40,253 participants and 1,095 events. The simple age-sex-PRS model includes fewer covariates than primary Model 3 and is interpreted separately from the fully adjusted estimates.

In primary-sample fivefold cross-validation, mean C was 0.84628 without OCN and 0.84642 with OCN (mean paired ΔC = 0.00014; fold-specific changes, −0.00035 to 0.00038; Supplementary Table S13). This small change provides no evidence of useful incremental discrimination. It does not assess calibration, decision utility or external validation. Age- and sex-stratified comparisons in Supplementary Tables S6, S7 use separate available-case populations and are descriptive.

After exclusion of baseline stroke, the all-cause dementia HR was 1.049 (95% CI, 0.983–1.118; *p* = 0.148) (*N* = 39,582; 1,057 events); VaD had HR 1.139 (95% CI, 0.989–1.311; *p* = 0.071) (219 events). Recruitment-center stratification yielded all-cause HR 1.047 (95% CI, 0.983–1.114; *p* = 0.155) and VaD HR 1.142 (0.995–1.310; *p* = 0.059). The White-restricted analysis gave all-cause HR 1.056 (0.990–1.127; *p* = 0.100; *N* = 37,828; 1,063 events; Supplementary Table S25D).

Two- and five-year landmark analyses retained the primary Model 3 specification (Supplementary Table S15A and Supplementary Figure S7). All-cause OCN HRs were 1.039 (95% CI, 0.975–1.107; *N* = 39,981; 1,067 events; *p* = 0.238) and 1.038 (0.971–1.110; *N* = 39,321; 965 events; *p* = 0.274), respectively. VaD after the five-year landmark had HR 1.167 (1.008–1.351; 202 events; *p* = 0.038), which remains nominal and exploratory. In matched complete records, adding baseline reaction time changed the OCN HR from 1.054 to 1.053 (*N* = 39,885; 1,072 events), and adding fluid intelligence changed it from 1.008 to 1.005 (*N* = 13,210; 279 events; Supplementary Table S15B). Supplementary Table S25 summarizes the main cognitive complete-record results, measured baseline molecular sensitivity and White-restricted Cox analysis. These checks do not eliminate a long preclinical disease period or residual confounding.

### 3.4 Systemic associations of OCN with bone mineral density and genetic risk variants

Skeletal and PRS associations were adjusted for age, sex, BMI and recruitment center (Supplementary Table S24). The baseline heel-BMD coefficient was −0.103 per SD of OCN for standardized BMD. Later-visit DXA coefficients are reported separately. Assay batch was unavailable and was not included as a covariate.

The Elastic Net composite-phenotype analysis included 7,923 measured complete records, 225 incident dementia events and 70 positive composite profiles (Supplementary Table S10 and Supplementary Figure S8). The tenfold internal AUC was 0.739 (fold SE, 0.024), with 42 nonzero coefficients at lambda.min = 0.004084. This is a partly OCN-defined composite and cannot establish dementia-specific predictive utility or causal protein effects. Elastic Net features did not contribute to the pathway foreground.

Standard and Enhanced genetic-score associations are distinguished by their field names in Supplementary Table S24 and Supplementary Figure S9. Phased APOE ε4 status, Standard AD PRS, and Enhanced apolipoprotein A1/B scores are separate variables. Cognitive and Cox models used the phased APOE carrier variable. A validated APOE variable was unavailable for MRI models, which did not adjust for APOE. Skeletal and PRS coefficients are displayed in Supplementary Figure S9.

Cognitive–dementia and MRI–dementia comparisons are shown in Supplementary Figures S10, S11. Their populations, scaling, and measurement timing differ from the baseline cognition and structural MRI analyses, and their full covariate and censoring specifications were incompletely documented. They are interpreted descriptively and do not establish prospective causal mediation.

### 3.5 Exploratory path models and measurement timing

The baseline path analysis included 24,144 participants for reaction time and 24,359 for pairs matching. In the specification with BMD as X and OCN as M (Supplementary Figure S12A), the statistical path product a × b was 0.00241 (95% bootstrap CI, 0.00109 to 0.00382) for reaction time and 0.00172 (0.00033 to 0.00308) for pairs matching. In the alternative specification with OCN as X and BMD as M (Supplementary Figure S12B), the products were 0.00004 (−0.00131 to 0.00124) and 0.00094 (−0.00039 to 0.00222), respectively. All variables were measured at the initial assessment. Differences between these model-based products do not identify the biological direction of influence or establish a compensatory mechanism. Higher reaction time and more pairs-matching errors indicate poorer test performance; the products should not be interpreted as fractions of cognitive benefit explained. We therefore report standardized coefficients and their uncertainty rather than mediated percentages. Fluid intelligence and numeric memory were omitted because the selected baseline BMD field did not overlap with their baseline observations in the analytical dataset.

Supplementary Figure S12C summarizes the measurement schedule. OCN and the cognitive measures used in the baseline path analysis were measured at the initial visit. MRI was obtained at later visits, and exact imaging dates could be recovered for only part of the imaging subcohort. Among 7,010 participants with i2 hippocampal measurements in the supplied incident cohort, 18 had dementia first recorded after the initial assessment: four had a confirmed MRI before their first report, one had a report before the i2 imaging programme began, and the ordering was unresolved for 13 (Supplementary Table S2).

### 3.6 Exploratory multi-omics associations of circulating OCN

In baseline metabolomics, 19,840 participants (44.3%) had no measurements for any of the 251 metabolites, while 24,928 had a measured profile; 2,319 of those profiles had at least one missing feature. Complete observed profiles were available in 22,609 participants. In proteomics, every participant had some baseline measurements, but none had all 2,922 non-OCN columns observed. The availability-filtered 890-protein complete matrix included 14,109 participants. No whole unmeasured profile or isolated missing value was filled in the molecular analyses (Supplementary Table S8 and Figure 4).

**Figure 4:**
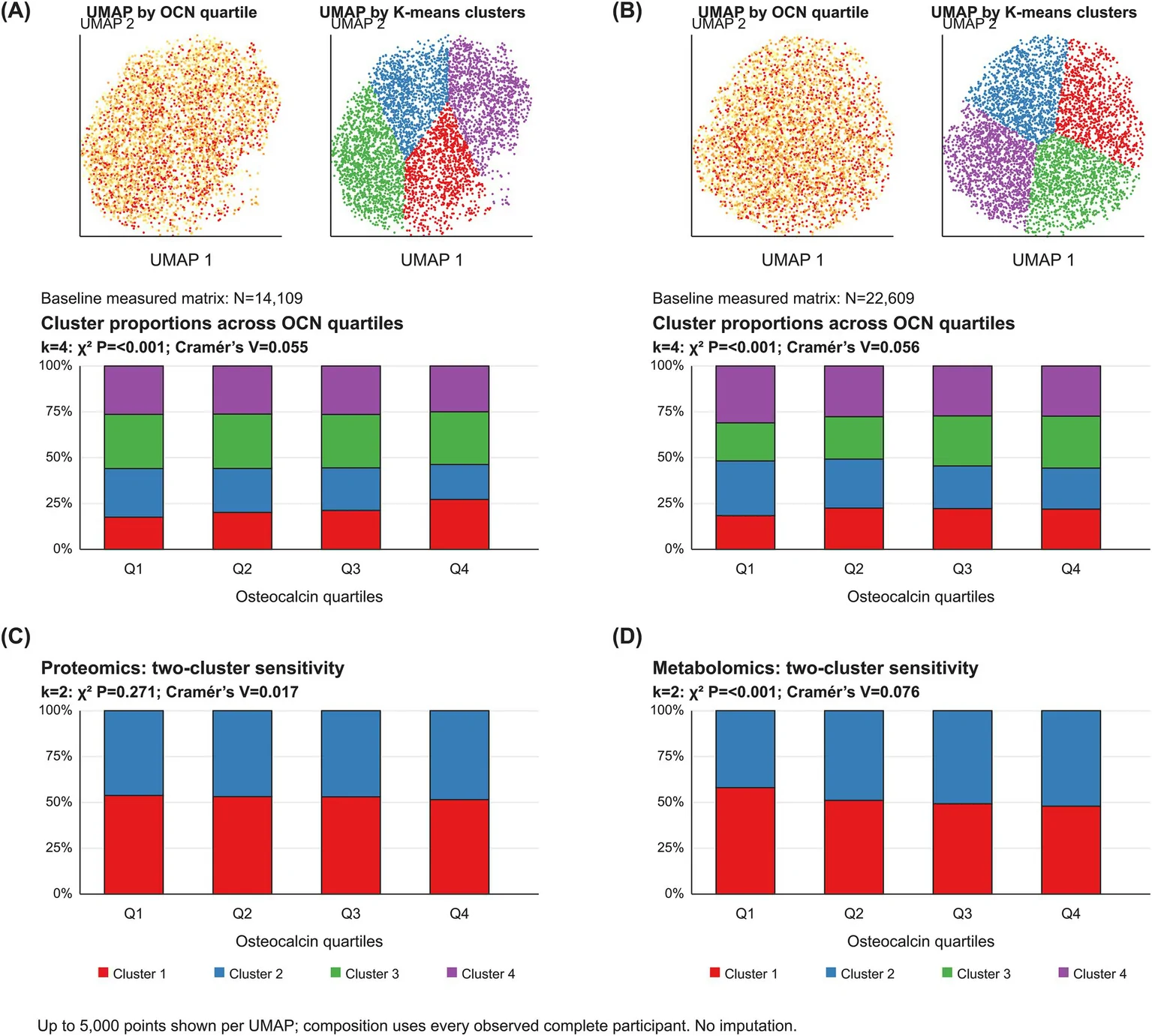
Baseline measured-only molecular UMAP and cluster-number sensitivity. **(A,B)** Proteomic and metabolomic UMAP projections colored by baseline OCN quartile or by the exploratory *k* = 4 K-means assignments, followed by cluster proportions within each OCN quartile. The protein matrix contains 14,109 complete participants across 890 features retained at ≥90% baseline availability; no participant had every one of the 2,922 non-OCN protein features measured. The metabolite matrix contains 22,609 participants with all 251 baseline metabolites observed. Entire unmeasured metabolite profiles and isolated missing values were excluded from these complete matrices; no later visit or imputation supplied a missing baseline value. Up to 5,000 points are displayed in each projection, while composition tests use every complete participant. **(C,D)** Cluster proportions within OCN quartiles for *k* = 2. UMAP used scaled features, 30 neighbors, min_dist = 0.3, 200 epochs, and seed 42; K-means used the Lloyd algorithm, 25 starts, and seed 123. Chi-square *p*-values and Cramér’s *V* are descriptive. The protein quartile-composition result was not significant at *k* = 2, so the *k* = 4 association should not be called invariant to cluster number. WGCNA assignment is summarized in Supplementary Table S9; parameter and bootstrap diagnostics are reported in Supplementary Figure S13 and Supplementary Table S28. Algorithm stability does not establish reproducible biological states.

The WGCNA Grey designation (Supplementary Table S9) indicates that OCN was not assigned to a module under the network settings. It is not evidence of a distinct co-expression process or absence of collinearity. The aggregate molecular inputs combined visits or incorporated filled values, limiting comparison with the observed baseline correlation screens.

The observed-pair protein correlations with OCN were 0.535 for CHAD (*N* = 43,242), 0.394 for SPP1 (*N* = 43,878), and 0.468 for COL1A1 (*N* = 14,525); age- and sex-adjusted partial rank correlations were 0.527, 0.386 and 0.460, respectively. Glucose was essentially uncorrelated with OCN (ρ = 0.00054; *p* = 0.933; *N* = 24,882; adjusted ρ = −0.0102; *p* = 0.107). Leucine, isoleucine and valine had unadjusted correlations of −0.0517, −0.0270 and −0.0444, respectively. After age and sex adjustment, correlations were −0.0146, −0.00003 and −0.0170; none survived Bonferroni correction across 251 metabolites (Figure 5 and Supplementary Tables S25B, S26). Baseline correlations for eight selected proteins are summarized in Supplementary Table S27.

**Figure 5:**
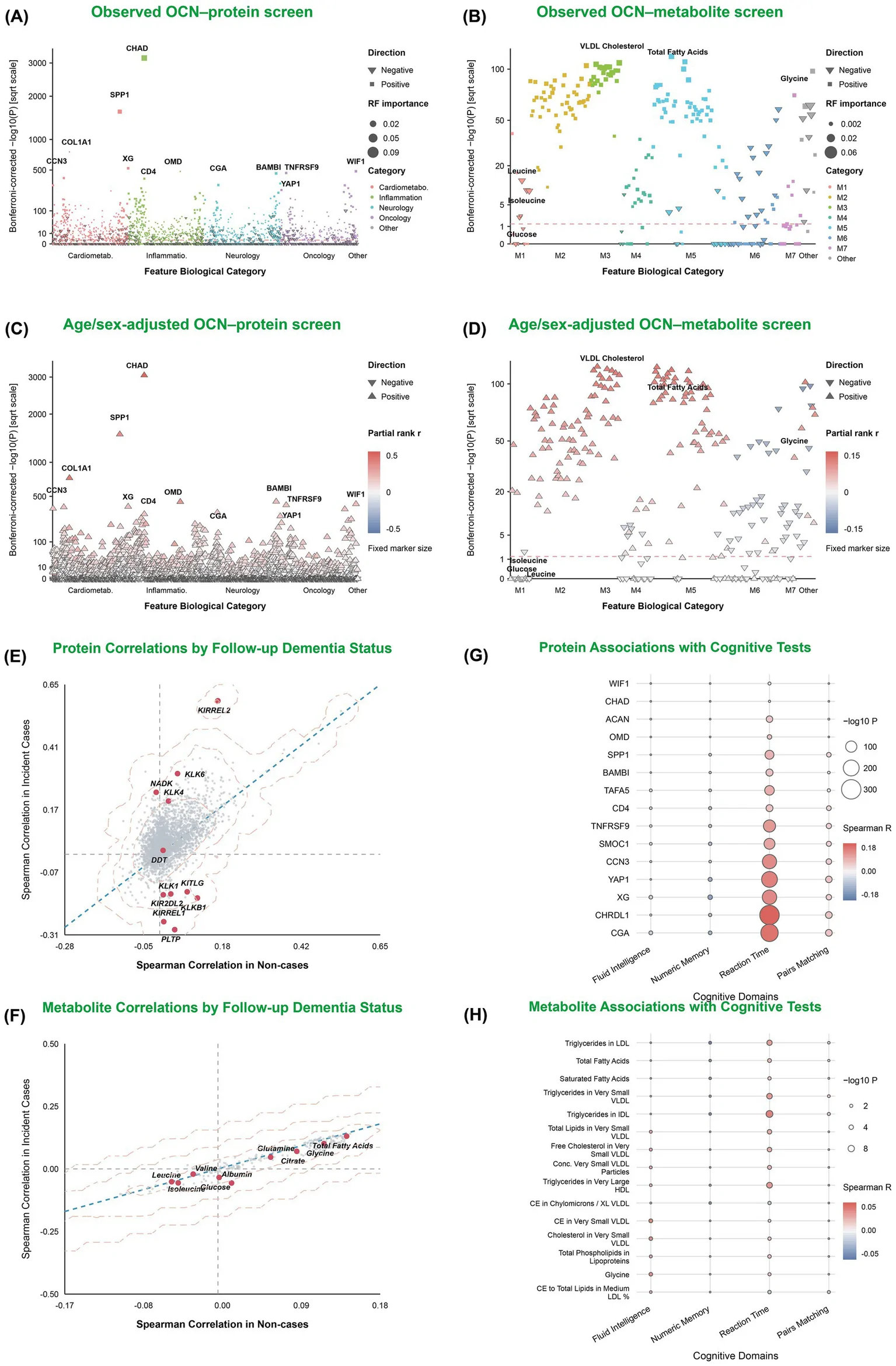
Exploratory baseline OCN-related molecular screens and cognitive associations. **(A,B)** Observed-pair OCN–protein and OCN–metabolite Spearman screens. The ordinate shows −log10 panel-wise Bonferroni *P* on a square-root display scale; all 2,922 non-OCN protein features and 251 metabolites define their respective correction denominators. Marker shape indicates association direction, color identifies the supplied biological category, and size displays nonnegative observed-matrix Random Forest importance for prediction of baseline OCN. Features outside the availability-filtered RandomForest matrix use the minimum marker size. This target is OCN, not dementia. M1–M7 are source metabolite-category labels, and other denotes an unmapped source feature. **(C,D)** Age- and sex-adjusted partial rank screens describe covariate-adjusted correlations. Triangles indicate correlation direction, color indicates partial rank correlation, and marker size is fixed; no mediated percentage or biological contribution is encoded. Correction again covers the full protein or metabolite panel. **(E,F)** OCN–feature correlations stratified by subsequent recorded dementia status in the longitudinal sample. Each point is a feature; the dashed diagonal denotes equal correlations. Highlighted labels and density contours are descriptive and do not test between-group differences. Non-case status denotes no recorded event before censoring, not lifetime disease freedom. **(G,H)** Selected baseline molecular–cognitive Spearman correlations, retaining 15 selected protein and 15 selected metabolite features. Bubble color indicates correlation direction and magnitude, and area represents nominal −log10 *P*. The parallel age- and sex-adjusted partial-rank sensitivity and all 120 pair entries are reported in Table S26C. Longer reaction time and more pairs-matching errors indicate poorer performance; higher fluid intelligence and numeric memory indicate better performance. These selected checks do not provide study-wide multiplicity control or establish mediation. No baseline value was filled by imputation or a later visit. Full numerical screen results accompany the analysis files, with selected results and cognitive pairs in Supplementary Tables S25, S26. OCN, osteocalcin; RF, Random Forest; CE, cholesterol esters; PL, phospholipids; FA, fatty acids; MUFA, monounsaturated fatty acids; PUFA, polyunsaturated fatty acids; VLDL, very-low-density lipoprotein; LDL, low-density lipoprotein; HDL, high-density lipoprotein.

Both two- and four-cluster observed-only solutions were evaluated (Supplementary Figure S13 and Supplementary Table S28). Quartile-composition results were Proteomics, *k* = 2: Cramér’s *V* = 0.017, *p* = 0.271; Proteomics, *k* = 4: Cramér’s *V* = 0.055, *P* = <0.001; Metabolomics, *k* = 2: Cramér’s *V* = 0.076, *P* = <0.001; Metabolomics, *k* = 4: Cramér’s *V* = 0.056, *P* = <0.001. Cluster-number, UMAP-grid and full-pipeline bootstrap diagnostics are reported without interpreting numerical stability as biological reproducibility. Random Forest predicted OCN in the measured complete samples, not dementia. Proteomic fivefold CV *R*^2^ was 0.262 and metabolomic CV *R*^2^ was 0.058 (Supplementary Table S29); these conditional-sample performances do not validate a clinical dementia biomarker.

Selected measured baseline molecular–cognitive Spearman correlations are displayed in Figures 5G,H, for 15 selected proteins and 15 selected metabolites. The corresponding age- and sex-adjusted partial rank sensitivity is reported in Supplementary Table S26C. All 120 selected checks are descriptive, their *p-*values are nominal, and they do not establish clinical prediction, mediation, or study-wide multiplicity control.

Outcome-stratified baseline correlations are displayed in Figures 5E,F. For COL1A1, correlations were 0.423 in 403 incident cases and 0.469 in 13,860 non-cases; for leucine, they were −0.051 in 728 cases and −0.051 in 23,718 non-cases. Differences are descriptive, were not formally tested and should not be called dementia-specific reprogramming. The non-case label denotes no event before censoring, not lifetime freedom from dementia.

With the baseline, age- and sex-adjusted foreground and no Elastic Net additions, four KEGG and 18 Reactome pathways survived BH correction (Supplementary Figure S14 and Supplementary Table S30). The leading KEGG terms were cell adhesion molecule interaction (*q* = 0.000014), complement/coagulation cascades (*q* = 0.000032), and cytokine–cytokine receptor interaction (*q* = 0.000051). Reactome included complement cascade (*q* = 2.39 × 10^−7^). These are enrichment descriptions of plasma associations, not evidence of dementia-related vascular injury. In the independent ST000046 plasma comparison, the BCAA score difference for AD versus controls was −0.104 standardized units (95% CI, −0.709 to 0.501; *p* = 0.727), and all three individual AD-control BCAA comparisons had BH *q* = 0.655. This small external cohort did not measure OCN and cannot validate OCN–BCAA coupling.

## 4 Discussion

This prospective cohort study examined baseline relative circulating OCN in relation to incident dementia and characterized its cognitive, imaging, skeletal, and molecular associations. The principal finding was the absence of a clear association with incident all-cause dementia after full adjustment (HR per SD, 1.052; 95% CI, 0.988–1.120; *p* = 0.117). In the same primary prospective sample, adding OCN changed the mean fivefold cross-validated C-index from 0.84628 to 0.84642 (ΔC = 0.00014). This small change does not establish useful incremental prediction. The confidence interval remains compatible with modest associations, so the result should not be interpreted as proof of biological inactivity. The nominal VaD association did not survive correction across the three secondary outcomes and weakened after excluding baseline stroke or accounting for recruitment center. Temporal variation in the AD association further cautions against treating a single hazard ratio as a complete summary. These primary results frame the interpretation of the exploratory phenotype and molecular analyses.

Earlier human studies addressed different populations, outcomes, and OCN measurements. Bradburn et al. (2016) examined 225 healthy older and 134 younger adults, with positive executive and global cognitive associations in older women. Puig et al. (2016) studied 24 obese adults and 20 healthy volunteers and reported associations of lower serum OCN with brain microstructural changes and worse cognitive performance. Bu et al. (2020) found higher plasma and CSF OCN in MCI and AD dementia, associations with CSF ATN biomarkers, and poorer cognition in a cross-sectional clinical sample. Luckhaus et al. (2009) likewise reported higher plasma OCN in mild AD than in cognitively normal controls, alongside increased markers of bone catabolism; the same pattern was not evident in MCI. These observations are heterogeneous and do not directly test incident dementia in an initially community-based population. Age, sex composition, disease status, specimen type, and calibrated clinical assays differ from the relative plasma Olink NPX signal used here, limiting comparison of the direction and magnitude of associations.

Casaletto et al. (2025) examined a CSF composite combining OCN/BGLAP and CSF2 with inversely weighted CCL2, CCL11, and B2M. Higher composite values were associated with slower clinical decline in a longitudinal cohort with autosomal dominant FTD and with less severe outcomes in a separate cross-sectional sporadic AD cohort. Associations for individual proteins varied by cohort. These findings support investigation of combined brain-aging signals, but do not validate isolated blood OCN as a predictor of incident dementia in the general population.

Mortality studies provide an additional comparison. Yeap et al. (2012) studied 3,542 men aged 70–89 years and reported higher mortality at both extremes of total OCN after adjustment. Lerchbaum et al. (2013) studied 2,271 men referred for coronary angiography and described a U-shaped association, but the high-OCN association lost statistical significance after multivariable adjustment; the low-OCN association persisted. Mortality differs from dementia, and these selected male populations differ from the present cohort. These findings cannot establish that higher relative OCN is generally harmful or that its prospective dementia association is monotonic. The continuous OCN estimate is therefore interpreted alongside exploratory quartile comparisons.

Preclinical work provides biological motivation for investigating bone-derived signals and brain function. Maternal and offspring OCN pools have been linked to brain development and function, and GPR158 has been implicated in OCN-related regulation of cognition in experimental models (Oury et al., 2013; Khrimian et al., 2017). Liu et al. (2024) and Zhang and Zhang (2024) review bidirectional bone–brain communication in AD, including bone-derived hormones, inflammatory processes, and effects of brain pathology on skeletal homeostasis. These reviews organize mechanistic hypotheses and possible therapeutic directions; they do not constitute clinical trials of OCN or validation of a circulating dementia biomarker. The experimental literature summarized by Obri et al. (2018) likewise provides a rationale for further investigation, but does not demonstrate a neuroprotective or compensatory endocrine response in this observational cohort.

Genetic-instrument studies address a different exposure contrast from a single circulating NPX measurement. Liu et al. (2023) reported a potentially protective genetically proxied OCN–AD association. Guo et al. (2024) reported lower AD odds with genetically predicted higher blood OCN in two outcome datasets; the estimates attenuated toward the null after multivariable adjustment for fasting glucose and total cholesterol, which the authors interpreted as potential metabolic mediation. This interpretation depends on valid instruments and multivariable MR assumptions. It neither establishes that metabolic mediation occurred in our cohort nor resolves differences between lifelong genetic exposure, assay-specific circulating signals, and clinical disease states. Our associations may reflect shared skeletal, vascular, and metabolic determinants as well as possible OCN biology.

Fully adjusted baseline cognitive associations were weak. In C3, the fluid-intelligence coefficient was close to zero, and the nominal reaction-time association (*β* = −0.0022 for ln[score+1] per SD OCN; *p* = 0.022) did not survive Holm correction across four domains (adjusted *p* = 0.090). Numeric memory and pairs matching likewise showed no clear fully adjusted associations. Fluid intelligence changed from a positive coefficient in C1 to approximately zero in C2 and C3, indicating sensitivity to socioeconomic and lifestyle adjustment. These findings do not support a broad claim of cognitive benefit. The negative-binomial sensitivity for pairs-matching errors addresses the count distribution without establishing temporal direction.

The repeated-visit cognitive models did not identify adequately sized classes for interpretable OCN class-membership analyses. They included 4,438 participants for fluid intelligence, 744 for numeric memory, and 9,005 for reaction time, with at least two distinct visits. Although BIC favored some multiple-class solutions, each contained a class below the prespecified 5% adequacy threshold. The smallest three-class groups comprised 0.29%, 2.02%, and 2.57% of the respective samples. High assignment probabilities do not make very small classes a reliable basis for subsequent regression. Class-membership associations were therefore not estimated for these solutions. This limitation concerns model adequacy and does not establish absence of an OCN relationship with cognitive change. Unequal visit intervals, limited repeat observations, and measurement variability constrain that question.

Skeletal and genetic associations provide additional physiological context. Baseline OCN was inversely associated with measured heel BMD, while associations with PRS were small and depended on the score used. Standard AD PRS, Enhanced AD PRS, and Enhanced apolipoprotein A1/B PRS are distinct variables; a PRS association is also distinct from adjustment for phased APOE ε4 carrier status. These observations may reflect shared determinants of bone and systemic health, but they do not establish a pathological high-turnover state or endocrine feedback. In the concurrent baseline path models, OCN, heel BMD, and cognition were measured at the same visit. A nonzero bootstrap-supported path product, or a difference between two fitted arrow directions, cannot establish a biological sequence. The baseline path products describe statistical associations and do not quantify a biological share of cognitive benefit or dementia risk.

The measured-only molecular screen confirmed substantial OCN correlations with CHAD, SPP1, and COL1A1; their age- and sex-adjusted partial rank correlations were 0.527, 0.386, and 0.460, respectively. COL1A1 was measured in only 14,525 participants, illustrating why a feature-specific observed-pair analysis can differ from a filled complete matrix. These associations justify follow-up of bone and extracellular-matrix biology, but plasma correlations do not identify tissue origin, blood–brain barrier injury, or a causal peripheral-to-brain pathway. Large plasma-proteomic dementia studies provide relevant context for exploratory pathway work (Walker et al., 2021; Walker et al., 2023), but overlap with a dementia-related protein literature does not establish dementia specificity in our OCN screen. The WGCNA Grey assignment denotes nonassignment under the chosen network settings; it does not demonstrate a distinct co-expression process or independence from other proteins. Random Forest performance refers to prediction of baseline OCN, and the Elastic Net target was partly defined by OCN itself; neither analysis constitutes validation of an independent dementia classifier.

The glucose and lipid findings must be interpreted against the numerical baseline results. Glucose was essentially uncorrelated with OCN in the observed-pair screen (ρ = 0.00054; *p* = 0.933), and its adjusted partial correlation was small (ρ = −0.0102; *p* = 0.107). The weak inverse unadjusted correlations with leucine, isoleucine, and valine attenuated after adjustment for age and sex, and none survived Bonferroni correction across all 251 metabolites. Selected lipid correlations remain descriptive rather than evidence of an OCN-driven metabolic state. Case/non-case correlation plots characterize participants according to recorded follow-up outcomes; their differences were not formally tested and should not be termed dementia-specific reprogramming. The measured, age- and sex-adjusted pathway analysis identified extracellular-matrix, cell-adhesion, cytokine, and complement-related terms, but did not demonstrate enrichment of BCAA catabolism. In the independent ST000046 cohort, the standardized BCAA-score difference for AD versus cognitively normal participants was −0.104 (95% CI, −0.709 to 0.501; *p* = 0.727), and the individual BCAA comparisons were not significant after correction. This small external cohort had no OCN measurements and cannot directly validate OCN–BCAA coupling.

MRI results were dependent on both model specification and participant selection. In the larger M2 samples, the nominal coefficients for total hippocampal volume and WMH were 0.0035 and 0.0339 per NPX unit for ln(volume). However, among the same 1,969 hippocampal participants, M2 was already near zero before PRS was added (*β* = 0.0002; *p* = 0.949); the corresponding M2 + PRS and M3 estimates were also near zero. In the same 1,926 WMH participants, M2, M2 + PRS, and M3 had coefficients of 0.0329, 0.0302, and 0.0292, with wide confidence intervals. These matched-sample results do not support a specific effect of adding genetic covariates. Missing PRS accounted for much of the reduction from the larger M2 samples: 4,900 of 6,869 participants for most volumes and 4,781 of 6,707 for WMH lacked at least one required score. Matched-sample comparisons address changing composition between fitted models but do not remove selection bias. Head-size, imaging-center, and blood-to-MRI interval variables were unavailable for the full analysis sample, and scan-to-diagnosis order was incompletely resolved. Baseline recruitment center, total brain volume, and head BMD were not used as substitutes for those imaging covariates.

Measurement differences are central to the comparison with earlier OCN studies. The UK Biobank proteomic resource reports Olink measurements as relative NPX values (Sun et al., 2023); PEA methodology establishes the analytical principle (Assarsson et al., 2014). Enroth et al. initially used relative PEA signals for ovarian-cancer discovery and subsequently developed an 11-protein test reporting absolute concentrations (Enroth et al., 2019). That assay-development study is not evidence for OCN prediction of dementia, and its calibrated final test should not be equated with the NPX exposure analyzed here. The available BGLAP assay information does not establish isoform-specific detection or equal quantitative recovery of carboxylated and undercarboxylated OCN. Their ratio cannot be derived from one NPX value. Vitamin K influences OCN carboxylation, while CKD and hemodialysis can alter circulating isoforms and their interpretation (Gundberg et al., 2012; Nagata et al., 2015). Relative OCN abundance should therefore not be equated with endocrine bioavailability or directly interpreted as osteoblast activity and bone turnover. This limitation concerns interpretation of the exposure itself, rather than merely the precision of a regression estimate.

Renal function remains an important unmeasured potential confounder. Impaired kidney function may influence circulating OCN through altered clearance and changes in bone metabolism (Gundberg and Weinstein, 1986), and kidney function and albuminuria have been associated with incident dementia in UK Biobank (Yeh et al., 2023). Serum creatinine, cystatin C, and eGFR were unavailable in the analytical dataset, so we could not determine whether direct renal adjustment would attenuate, strengthen, or otherwise alter the associations. Adjustment for hypertension, diabetes, or recruitment center does not substitute for measured renal function. Given the small magnitude and model dependence of several findings, the results cannot be described as independent of renal confounding.

The study’s strengths include the prospective ascertainment of dementia, explicit specification of the primary adjustment models, comparisons of MRI models in the same participants, and observed-pair molecular screens covering all baseline features. The main inferential limits are a single baseline OCN measurement, outcome ascertainment from routine records, selection into measured molecular and imaging subsets, unavailable assay batch and genetic principal components, and multiple exploratory analyses. There was no all-feature complete protein matrix; the 890-feature multivariate analysis consequently represents an availability-filtered subset rather than the entire protein panel. Internal cross-validation and algorithm stability checks cannot establish external predictive utility or biological reproducibility. Figure 6 therefore summarizes the evidence in the anatomical context of the measured phenotypes without asserting an endocrine cascade. Replication with calibrated isoform-aware assays, measured renal function, appropriate imaging covariates, and prospectively aligned repeated measurements is needed to evaluate the competing explanations and any reproducible prognostic value.

**Figure 6:**
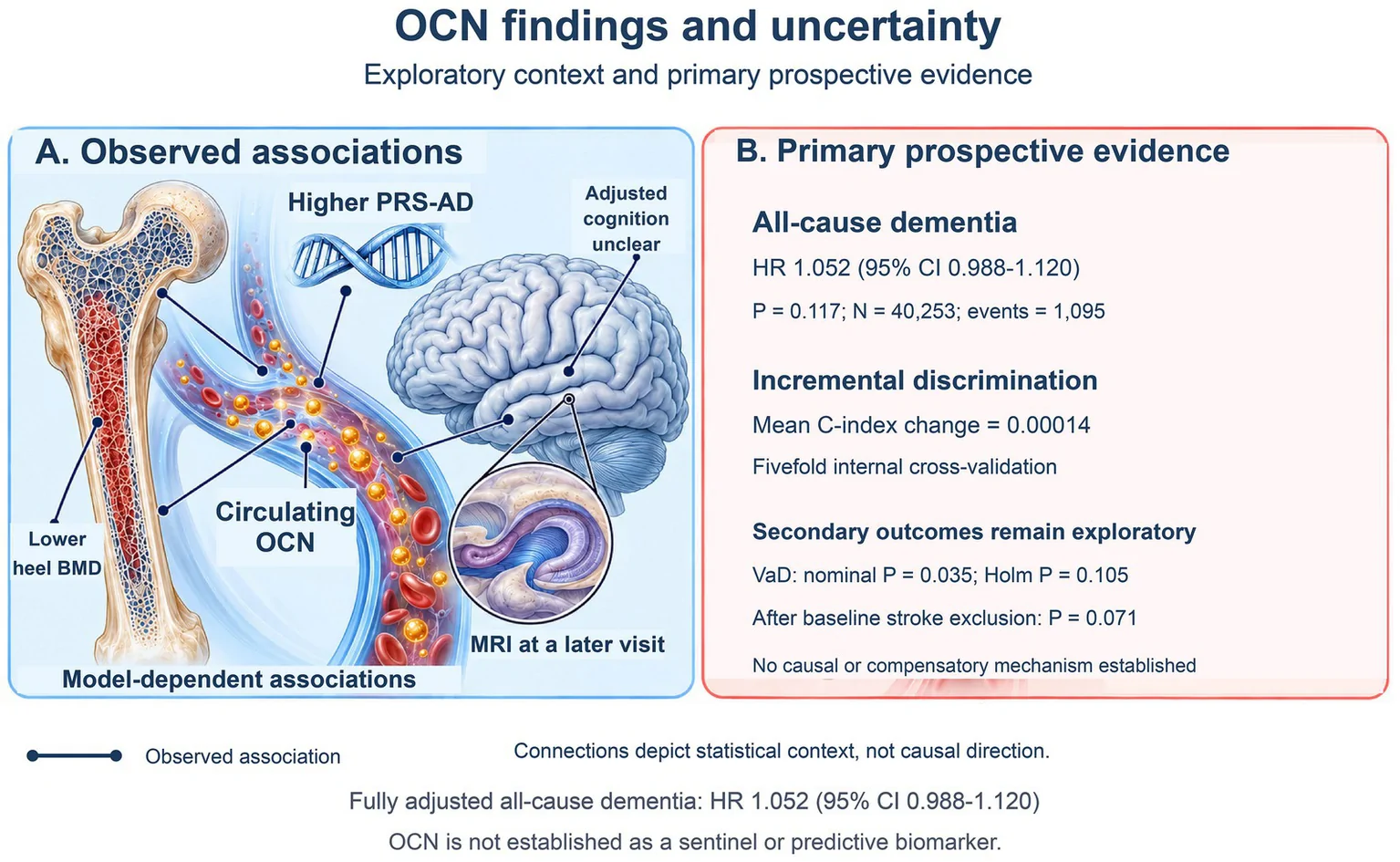
Summary of phenotypic associations and prospective dementia results. **(A)** Anatomical context for the observed OCN, skeletal, genetic, cognitive, and later MRI associations. Connectors depict statistical associations and do not establish causal direction, tissue origin, endocrine feedback, or a compensatory mechanism. Results depend on their sample, measurement visit, and adjustment model; the fully adjusted cognition results and matched MRI comparisons are provided in Figure 2, Supplementary Figure S1, and Supplementary Tables S3, S4. **(B)** Summary of the primary prospective evidence: the fully adjusted all-cause dementia HR per SD OCN is 1.052 (95% CI, 0.988–1.120; *p* = 0.117), and the same-sample fivefold change in mean *C*-index is approximately 0.00014. The nominal VaD result did not survive secondary-outcome correction and weakened after excluding baseline stroke. The retained anatomical visual framework contextualizes the exploratory associations without asserting a bone-to-brain endocrine cascade or clinically validated sentinel biomarker.

## 5 Conclusion

Baseline relative OCN was not clearly associated with incident all-cause dementia after full adjustment and did not show useful incremental discrimination in the available prospective sample. Subtype, cognitive, imaging and measured baseline molecular findings remain exploratory. They do not establish causal mediation, a compensatory endocrine response or clinical biomarker utility. Residual confounding by renal function remains unresolved because renal measures were unavailable. Replication with measured renal function and consistent prospective timing is required.

## Data Availability

The original contributions presented in the study are included in the article/Supplementary material, further inquiries can be directed to the corresponding author.

## Footnotes

1
http://www.ukbiobank.ac.uk

## Glossary

AD: Alzheimer’s Disease
BMD: Bone Mineral Density
BMI: Body Mass Index
CHAD: Chondroadherin
CI: Confidence Interval
COL1A1: Collagen alpha-1(I) chain
CSF: Cerebrospinal Fluid
DAM: Disease-Associated Microglia
FSH: Follicle-Stimulating Hormone
FTD: Frontotemporal Dementia
HR: Hazard Ratio
LDL: Low-Density Lipoprotein
MRI: Magnetic Resonance Imaging
OCN: Osteocalcin
OR: Odds Ratio
PET: Positron Emission Tomography
PRS: Polygenic Risk Score
RCS: Restricted Cubic Spline
RF: Random Forest
SD: Standard Deviation
sHR: Subdistribution Hazard Ratio
SPP1: Secreted Phosphoprotein 1 (Osteopontin)
UKB: UK Biobank
UMAP: Uniform Manifold Approximation and Projection
VaD: Vascular Dementia
VLDL: Very Low-Density Lipoprotein
WMH: White Matter Hyperintensities
