# Supplementary material for: Circulating osteocalcin and incident dementia in the UK Biobank: a prospective cohort and exploratory multi-omics analysis
Source: Front Aging Neurosci. 2026 Oct 26;18:1974960. doi: 10.3389/fnagi.2026.1974960 (PMC13745723; doi:10.3389/fnagi.2026.1974960)
Supplement: Supplementary file 1 [file Data_Sheet_1.pdf]

Additional File

Supplementary methods figures and table titles

Supplementary figure titles

Figure S1. Structural MRI associations across Models 1–3 in identical outcome-specific samples.

Figure S2. Exploratory PRS associations with recorded dementia outcomes.

Figure S3. Distribution and regression diagnostics for four cognitive domains.

Figure S4. Exploratory OCN–dementia spline models.

Figure S5. Relative OCN across cognitive performance groupings.

Figure S6. Baseline cognitive associations in Models C1–C3.

Figure S7. Landmark and descriptive competing-risk sensitivity comparisons.

Figure S8. Elastic Net coefficients for the baseline OCN–BMD–dementia composite profile.

Figure S9. Skeletal and PRS associations with baseline OCN.

Figure S10. Exploratory cognitive-dementia Cox associations with and without additional OCN adjustment.

Figure S11. Exploratory Cox associations of MRI phenotypes with all-cause dementia, with and without OCN adjustment.

Figure S12. Exploratory baseline association paths and measurement timing.

Figure S13. Measured-only clustering diagnostics.

Figure S14. OCN-related protein pathways and independent BCAA comparison.

Supplementary table titles

Table S1. Catalogue of UK Biobank field IDs and descriptions.

Table S2. Timing of cognitive and MRI measurements relative to first recorded dementia diagnosis

Table S3. Plasma OCN associations with structural MRI: available-case and matched-sample estimates, sample availability, and multiplicity correction.

Table S4. Baseline plasma OCN associations with cognition in Models C1–C3 and negative-binomial sensitivity analyses of pairs-matching errors.

Table S5. Simple sequential Cox models in the prospective cohort

Table S6. Exploratory subgroup associations of osteocalcin with subsequent all-cause dementia.

Table S7. Exploratory age- and sex-stratified associations in the full cohort.

Table S8. Missingness at baseline and in the longitudinal cohort

Table S9. OCN WGCNA assignments in aggregate molecular data

Table S10. Baseline composite-phenotype Elastic Net audit

Table S11. Plasma OCN and incident dementia: continuous and quartile Cox estimates, model precision, and log-rank comparisons.

Table S12. Primary Cox diagnostics and clinical sensitivities

Table S13. Primary-sample incremental discrimination

Table S14. Exploratory observed-record AIC-selected Cox model

Table S15. Two- and five-year landmark Cox analyses and exploratory adjustment for baseline cognitive performance.

Table S16. Variable coding and primary dementia outcome definitions.

Table S17. Participant characteristics of the full OCN cohort and longitudinal cohort.

Table S18. Participant characteristics by recorded dementia subtype in the full and longitudinal cohorts.

Table S19. Participant characteristics by availability of MRI and heel BMD measurements.

Table S20. Participant characteristics of participants with OCN and available cognitive measurements.

Table S21. Participant characteristics of participants with OCN and available structural MRI measurements.

Table S22. Repeated-visit cognitive model selection and class adequacy

Table S23. Exploratory linear and nonlinear associations of selected participant characteristics with relative plasma OCN.

Table S24. Recruitment-centre-adjusted associations of relative plasma OCN with skeletal phenotypes and polygenic risk scores.

Table S25. Complete-record and measured-baseline sensitivity analyses

Table S26. Baseline OCN–protein and OCN–metabolite screens: test coverage, selected correlations, and molecular–cognitive associations.

Table S27. Baseline correlations of relative plasma OCN with eight selected proteins, with age- and sex-adjusted sensitivities.

Table S28. Measured-only cluster composition and algorithm diagnostics

Table S29. Observed-only Random Forest prediction of baseline OCN

Table S30. OCN-associated protein pathway enrichment and independent BCAA comparisons among AD, MCI, and cognitively normal participants.

Supplementary Methods

2.1 Study population and sample

UKB is a large-scale prospective cohort study that recruited over 500,000 participants aged 37–73 years from 22 assessment centers across the United Kingdom between 2006 and 2010. The study protocol is available online (http://www.ukbiobank.ac.uk). This analysis was conducted under UK Biobank application number 190997. The study received ethical approval from the North West Multi-Centre Research Ethics Committee (11/NW/0382), and all participants provided electronic signed informed consent at recruitment.

In the present study, we focused on participants with available proteomic data from the UK Biobank Pharma Proteomics Project (UKB-PPP), which measured plasma protein levels using the Olink Explore 3072 platform. Because this study constitutes a retrospective analysis of an existing eligible cohort, no formal a priori sample-size

or statistical power calculation was performed; the analytical sample size was intrinsically determined by the availability of valid Olink data and clinical covariates. Following standard epidemiological protocols, we applied strict inclusion and exclusion criteria. Analytical samples were defined separately for each outcome and its required covariates; completeness across all phenotypes was not required for the primary longitudinal analyses. For the cross-sectional analyses, these eligible participants were included to assess baseline physiological associations. However, for the longitudinal survival analyses evaluating incident dementia risk, to minimize reverse causality and potential confounding from pre-existing neurological damage, we explicitly excluded participants who, at baseline, met any of the following criteria: (1) a prior diagnosis of dementia, Parkinson's disease, multiple sclerosis, or other severe neurodegenerative and demyelinating disorders (e.g., amyotrophic lateral sclerosis, Huntington's disease; detailed ICD-10 codes are provided in Table S1); or (2) a history of severe head injury or neurotrauma. The longitudinal cohort comprised 43,942 participants. All model-specific sample sizes refer to observed complete records. The cohort flow and analysis families are summarized in Figure 1.

2.2 Primary exposure

Olink BGLAP was measured as Normalized Protein eXpression (NPX) on a log2 scale. Primary Cox and baseline cognitive coefficients are per one SD of baseline OCN, standardized in the available prospective cohort. MRI coefficients remain per one NPX unit for ln(volume); no additional log or Z transformation was applied to MRI OCN. Molecular results are Spearman correlations or age- and sex-adjusted partial rank correlations. These quantities and scales are labeled separately.

We refer to the Olink BGLAP signal as relative circulating OCN abundance. NPX is not an absolute concentration. The available assay information and cohort data do not establish isoform-specific detection or the relative antibody response to carboxylated and undercarboxylated OCN. We therefore do not interpret this measurement as an isoform-specific concentration, a validated quantitative total-OCN assay, or a measure of the ucOC/cOC ratio.

2.3 Primary outcome

The primary outcome was incident all-cause dementia. Secondary analyses examined Alzheimer’s disease (AD), vascular dementia (VaD), and frontotemporal dementia (FTD). Outcomes were ascertained from linked UK Biobank algorithmic dementia reports and first-recorded ICD-10 diagnoses. AD used G30/F00 and the algorithmic AD report; VaD used F01 and the algorithmic VaD report; FTD used the algorithmic FTD report. The earliest available dementia date was used for ascertainment.

The follow-up period was calculated from the date of attending the assessment center to the date of diagnosis, death, or the end of follow-up (November 27, 2024), whichever occurred first.

2.4 Cognitive outcomes

The baseline cognitive analyses used initial-assessment (instance 0) scores in the available incident-dementia cohort, measured concurrently with baseline OCN. Fluid intelligence and numeric memory scores increase with better performance; reaction time and pairs-matching errors increase with worse performance. Valid scores were transformed as ln(x+1), with negative missing/refusal codes excluded. Pairs matching used round 1 only. Cognitive Models C1–C3 used the primary Cox covariate sets, without a survival outcome. Coefficients are changes in ln(score+1) per SD of baseline OCN. Outcome-specific complete records were used; neither scores nor covariates were imputed. These concurrent associations cannot establish temporal direction.

2.5 Neuroimaging phenotypes

The OCN–MRI associations in Figure 2B and Figure S1 used structural MRI measurements from the first imaging assessment (instance 2) in the full OCN cohort of 44,768 participants. No baseline neurological exclusions were applied to this descriptive MRI analysis, which is separate from the incident-dementia cohort. Repeat-imaging

measurements from instance 3 were not included in these regressions. We analyzed left, right, and total hippocampal volumes; grey matter, white matter, and total brain volumes; ventricular cerebrospinal fluid (CSF); left and right thalamic and amygdala volumes; and total white matter hyperintensity (WMH) volume. Total hippocampal volume was calculated by summing the left and right measurements before transformation. All MRI outcomes were natural-log transformed; non-positive volumes were treated as missing. The fields used were unnormalized volume measures in mm^3, not the UK Biobank head-size-normalized global-volume fields. Intracranial volume, the T1 head-size scaling factor, and imaging-centre information were unavailable in the MRI analytical dataset and were not included. Individual imaging dates were available only through a partial linkage used for Table S2; the blood-sampling-to-MRI interval was not included in these regressions. Baseline age and age-squared were included, but neither imaging age nor the interval was substituted by baseline age. MRI was obtained after the OCN measurement, and scan-to-diagnosis ordering could not be verified throughout the sample. These associations remain exploratory and are not interpreted as causal mediators of incident dementia.

2.6 Bone Mineral Density Assessment

Heel bone mineral density (BMD), measured by quantitative ultrasound at instance 0, was the primary skeletal measure. Later-visit DXA measurements were examined separately as exploratory phenotypes. In skeletal and PRS regressions, OCN and each outcome were standardized, and models adjusted for baseline age, sex, BMI, and recruitment centre. Assay batch information was unavailable and was not adjusted for. Later DXA associations are not concurrent baseline measurements.

2.7 Genetic Risk Assessment

Genetic-score specifications differed between analysis families. Primary Cox Model 3 used the Standard PRS for Alzheimer’s disease (AD). MRI Model 3 used Standard AD PRS (Field 26206), Enhanced apolipoprotein A1 PRS (Field 26208), and Enhanced apolipoprotein B PRS (Field 26209), entered as continuous scores. Enhanced AD PRS (Field 26207) was examined in separate exploratory associations and was not included in MRI models. Apolipoprotein A1/B scores are distinct from APOE ε4 carrier status. APOE ε4 status was not established in the MRI model dataset, and these models did not adjust for APOE. The bulk-genotyping indicator (Field 22418) was not used as an APOE genotype. Exploratory PRS–dementia comparisons are shown in Figure S2 and do not define the primary adjustment model.

2.8 Covariates

The primary prospective Cox analysis used the following progressive adjustment models. Model 1 was minimally adjusted for basic demographic factors: chronological age, age-squared, sex, and body mass index (BMI). Model 2 further incorporated socioeconomic status and lifestyle factors, including qualifications (education level, utilized as a robust proxy for cognitive reserve), Townsend deprivation index, smoking status, alcohol drinking frequency, and moderate physical activity, alongside ethnic background. Due to the vast majority of participants being of White ancestry and the extremely low number of incident dementia events across other individual ethnic groups, ethnic background was dichotomized as "White versus other" to maintain statistical power and model stability. Model 3 (Fully adjusted model) additionally accounted for genetic predispositions and core cardiometabolic comorbidities: the standard polygenic risk score for AD (PRS-AD), APOE ε4 carrier status, and a history of hypertension (I10), diabetes (E10-E11), and stroke or cerebrovascular disease (I60-I64).

MRI model definitions were specified separately from the primary Cox models. MRI Model 1 adjusted for baseline age, age-squared, sex, and BMI. MRI Model 2 additionally adjusted for qualifications, Townsend deprivation index, smoking status, alcohol drinking frequency, moderate physical activity, ethnic background, and baseline handedness; handedness was included for every MRI outcome. MRI Model 3 additionally included the three PRS variables specified in Section 2.7 and baseline diagnosis indicators for the exact ICD-10 codes listed in the Supplementary Methods. It therefore combined genetic-score and disease-history adjustment. An intermediate

Model 2 + PRS added only the three genetic scores. For each outcome, Models 1, 2, 2 + PRS, and 3 were compared in the identical Model 3 complete-case sample. Figure 2B presents Model 2 in its larger outcome-specific complete-case sample; Figure S1 and Table S3 report the matched-sample comparisons. No clinical, genetic, or MRI values were imputed.

Baseline cognitive associations were estimated using Models C1–C3 in the prospective cohort (Table S4). C1 includes age, age-squared, sex and BMI; C2 adds education, Townsend deprivation, smoking, alcohol, activity and White-versus-other ethnicity; C3 adds Standard AD PRS, phased APOE ε4 status and baseline hypertension, diabetes and stroke. These cognitive models differ from MRI Models M1–M3.

Sequentially adjusted Cox sensitivity models used the same prospective cohort, outcome dates and administrative cutoff as the primary analysis: unadjusted, age-adjusted, age-and-sex-adjusted, and age-sex-Standard-AD-PRS-adjusted models (Table S5). Subgroup comparisons in Tables S6 and S7 used different available-case populations and outcome definitions and are interpreted as descriptive comparisons rather than fully adjusted prospective estimates.

Renal function measures, including serum creatinine, cystatin C, and estimated glomerular filtration rate (eGFR), were not available in the analytical extract used for this study and were therefore not included in the adjustment models.

2.9 Exploratory Multi-Omics and Statistical Path-Model Screens

Molecular analyses used measured baseline features only. Protein data were taken from the baseline measurement block, with repeat-visit blocks excluded. Metabolites used columns labeled Instance 0. Repeated molecular measurements were not averaged, and later values did not substitute for missing baseline values. We screened 2,922 non-OCN baseline protein columns and 251 metabolites using feature-specific observed pairs. Whole-profile nonmeasurement was distinguished from isolated missing values (Table S8). No values were imputed in correlation screens, clustering, or Random Forest analyses.

For observed-only multivariate analyses, baseline features with at least 90% observed values among participants with a measured panel were retained, followed by complete-record selection across those features. This yielded 890 proteins in 14,109 participants and all 251 metabolites in 22,609 participants. There were no participants with every one of the 2,922 protein columns observed; thus, the complete protein matrix is an availability-filtered sensitivity analysis, not a complete-case replication of the entire panel. Scaling, UMAP (30 neighbours, min_dist=0.3; seed 42; 200 epochs), and K-means (Lloyd algorithm; nstart=25; maximum 1,000 iterations; seed 123) were fitted to each complete matrix. Both k=2 and k=4 were evaluated. Full-matrix UMAP parameter checks used 15/0.1 and 50/0.5. Twenty participant bootstrap resamples of a fixed 3,000-person evaluation subset fitted scaling, UMAP and clustering within each resample; ARI was evaluated in the unique resampled participants against the subset reference. Availability-filtered feature sets were held fixed. This tests algorithmic stability conditional on the measured sample, not biological reproducibility.

Random Forest regression predicted continuous baseline OCN from the observed complete molecular matrices, using ranger with 300 trees, mtry=floor(sqrt(number of features)), minimum node size 5 and permutation importance. Fivefold cross-validation trained each forest within its training fold; the target was OCN, not dementia. Every baseline feature was also tested using feature-specific observed Spearman correlation and partial rank correlation adjusted for age and sex. Panel-wise Bonferroni correction used 2,922 protein and 251 metabolite tests. WGCNA assignments based on aggregate molecular inputs are reported separately in Table S9; their measurement visits and missing-data processing differ from the baseline screens. Grey denotes nonassignment under the network settings and does not imply a distinct biological process or independence. Molecular correlations were not interpreted as fractions of dementia risk explained.

Baseline molecular correlations were also described separately in participants with a recorded incident dementia event by November 27, 2024 and those without an event by their censoring date in the prospective extract. These are outcome-stratified descriptions, not formal tests of correlation differences or dementia-specific mechanisms. No later molecular measurements or within-person transition were inferred.

Pathway over-representation used baseline proteins that survived age- and sex-adjusted panel-wise Bonferroni correction, intersected with the assay-background gene mapping. Elastic Net features were not added to the pathway foreground. The measured mapped background comprised 2,911 gene symbols (2,872 KEGG-mapped Entrez IDs); the foreground contained 1,011 symbols (1,001 Entrez IDs). One-sided hypergeometric tests were BH-adjusted across 321 eligible KEGG and 1,130 Reactome pathways using the official gene-set releases used for this analysis. Pathways with 5–500 background genes were tested.

Elastic Net was fitted separately in participants of the prospective cohort with measured baseline OCN, heel BMD and all 890 retained baseline protein features (N=7,923; 225 incident dementia events). The positive composite required OCN above the cohort median (NPX>0), BMD below its cohort median (0.527 g/cm²), and incident dementia before censoring; 70 participants met all three conditions. Binomial glmnet used alpha=0.5, standardized predictors, seed 20261007, ten class-stratified folds, AUC selection and lambda.min. This outcome partly incorporates OCN itself, so it is neither an independent dementia classifier nor evidence of causal drivers. Its selected proteins were excluded from pathway enrichment (Table S10).

Additionally, we explored BCAA patterns using the independent Mayo Clinic plasma metabolomics cohort ST000046 (n=15 each for Alzheimer's disease, mild cognitive impairment, and cognitively normal). Log2-transformed LC–MS intensities for leucine, isoleucine, and valine, alongside an equally weighted standardized composite BCAA score, were compared between groups using Welch's two-sample t-tests with BH correction. This cross-sectional analysis assessed the generalizability of the BCAA disease contrast independently of OCN.

2.10 Statistical analysis

Participant characteristics were summarized as means (SD), medians [interquartile ranges], or counts (percentages). Clinical models excluded unknown/refusal categorical codes without imputation or Unknown factor levels. Valid negative Townsend values were included. Qualifications [-7] denotes "None of the above"; unknown/refusal codes were excluded. Primary models used phased APOE data linked by participant ID from the full cohort. SNP-labelled dosage columns and the bulk-genotyping indicator were not used to infer APOE ε4 status. Genetic principal components were unavailable in the analytical dataset and were not included in the models. MRI-specific coding is detailed in Supplementary Methods 2A.

Baseline cognitive associations used C1–C3 linear regressions of ln(score+1) in observed complete records. A negative-binomial sensitivity analysis examined baseline pairs-matching error counts using the same C1–C3 covariates and per-SD OCN scale. Source distribution diagnostics are provided in Figure S3. The LCMM analysis used participants with observations at two or more distinct visits, with one value per domain per visit. Fluid intelligence, numeric memory and reaction time were transformed as ln(x+1) and scaled for fitting. Time was the visit index 0–3, not years, because individual follow-up visit dates were unavailable throughout the extract. Random-intercept, linear-visit hlme models with 1–3 classes were evaluated; two- and three-class fits used ten random starts, a 20-iteration search and up to 200 final iterations. Convergence code 1 was required. BIC, class size, posterior probabilities and entropy were reported. Multiple classes with any class below 5% were not used for subsequent class-membership regression. This is a model-adequacy rule, not proof of a single biological trajectory. Pairs matching was not used for trajectory inference because zero inflation and within-visit repeated rounds do not provide independent follow-up occasions.

Primary Cox models used the baseline covariate hierarchy, per-SD OCN and Efron ties. Schoenfeld tests were reported for every Model 3 term and globally. Because the all-cause model showed departures for age and alcohol

and the AD OCN term was non-proportional, sensitivities used flexible age splines, age-band/alcohol-stratified baseline hazards, and separate OCN effects during 0–5 and >5 years. Recruitment-centre-stratified Cox models and exclusion of baseline stroke were additionally fitted. Assay batch was unavailable in the extract. Model-specific N, events, precision and primary continuous OCN estimates are consolidated in Table S11; diagnostics and sensitivity results are in Table S12. Restricted cubic spline displays in Figure S4 are descriptive and are not substitutes for the continuous primary estimates.

Incremental discrimination was assessed in the primary Model 3 complete-case sample (N=40,253; 1,095 events) using the prospective outcome dates and administrative cutoff. Fivefold cross-validation compared models with and without OCN in identical training and test participants, using Harrell C with higher linear predictors denoting higher risk. Mean fold-specific C and the paired change are reported in Table S13. An exploratory bidirectional stepwise Cox model included heel BMD, used AIC, and had N=22,882 with 679 events (Table S14); no Unknown smoking or alcohol categories were fitted.

All-cause dementia was the single primary survival outcome. Holm correction was applied to the three Model 3 secondary outcomes (AD, VaD and FTD), and separately to four C3 cognitive tests. For MRI, BH correction was reported across 12 outcomes within each displayed model/sample family, while nominal P values were retained. Molecular screens used panel-wise Bonferroni correction and pathways used BH correction. Other model stages, subgroup, interaction, clustering, feature-selection and sensitivity analyses remain exploratory; correction within one family does not provide study-wide error control.

We fitted exploratory statistical path models using only initial-assessment (instance 0) measurements in the cohort. Heel BMD was taken from the numerical Heel bone mineral density field, without substitution by a T-score or a later-visit measure. OCN was taken from baseline Olink BGLAP. Cognitive outcomes were baseline reaction time and the number of incorrect matches in pairs-matching round 1. Fluid intelligence and numeric memory were not analyzed in these path models because neither had overlapping observations with the selected instance-0 Heel BMD field in the analytical dataset. Cognitive outcomes were transformed as $\log(x + 1)$, and OCN, BMD, and transformed cognition were standardized within each outcome-specific complete-case sample. Models adjusted for age, sex, BMI, Townsend deprivation index, and university-degree status. The same participants were used for the two alternative specifications within each cognitive outcome. These limited-adjustment, exploratory models do not establish temporal direction or causal mediation.

Two- and five-year landmark analyses retained the primary Model 3 specification and November 27, 2024 cutoff; follow-up restarted at each landmark in alive, uncensored, dementia-free participants. Primary cognition-adjusted checks are shown in Table S15. The White-restricted Cox sensitivity was fitted with the same clinical records, baseline exclusions, covariates and censoring date as the primary model; White-versus-other was removed because ethnicity was constant. The analysis included 37,828 complete records and 1,063 events. Competing-risk comparisons use a separate minimally adjusted specification and are interpreted descriptively. Analyses used R 4.6.1 and Python. Code and aggregate outputs are provided with the supplementary materials.

Supplementary Methods 2A. MRI analysis specification

Population and outcomes. The MRI analysis used the full 44,768-participant OCN extract, including participants with baseline recorded diseases; no neurological exclusions from the incident-dementia cohort were imposed. Each participant contributed the first imaging-visit measurement (instance 2), with no substitution from instance 3. Baseline OCN was olink_instance_0.bglap on its NPX scale. The MRI outcomes were left/right hippocampus (25019/25020), their sum, grey matter (25006), white matter (25008), total brain (25010), ventricular CSF (25004), left/right thalamus (25011/25012), left/right amygdala (25021/25022), and total WMH (25781). All MRI outcomes were transformed as ln(volume), with values ≤ 0 treated as missing. These were unnormalized mm^3 volume fields. Neither the head-size-normalized global-volume fields nor the T1 head-size scaling factor (25000) was used.

Covariate coding. Age, BMI, Townsend deprivation index, and days per week of moderate physical activity were continuous baseline variables. Age was entered as age plus age-squared, algebraically equivalent to the second-degree age polynomial in the recovered script. Sex, smoking status, alcohol drinking frequency, ethnic background, handedness, and qualifications were categorical. Qualifications were modeled using the recorded multi-response combinations as factor levels, with [-7] retained as "None of the above" (reference); [-3] and blank values were missing. Ethnicity retained the individual recorded categories, with British (1001) as reference, rather than the White-versus-other coding used in the primary Cox analysis. The reference codes were sex 0, smoking 0, alcohol 1, and handedness 1. Negative unknown/refusal codes in smoking, alcohol, physical activity, ethnicity, and handedness were set to missing. Negative Townsend values were retained. Clinical, PRS, and imaging values were not imputed.

Exact models. For each MRI outcome Y, MRI Model 1 was $\ln(Y) \sim \text{OCN\_NPX} + \text{age} + \text{age}^2 + \text{sex} + \text{BMI}$. MRI Model 2 added qualifications + Townsend deprivation index + smoking status + alcohol drinking frequency + moderate physical activity days/week + ethnic background + handedness. MRI Model 3 added Standard AD PRS (26206) + Enhanced apolipoprotein A1 PRS (26208) + Enhanced apolipoprotein B PRS (26209) + the separate baseline ICD-10 indicators listed below. PRS columns were selected by their exact names and entered as continuous supplied scores without additional standardization. Model 2 + PRS was an intermediate specification containing Model 2 and these three PRS variables, with no disease-history additions. This intermediate model was fitted in the same Model 3 complete-case participants to distinguish genetic-score additions from subsequent disease-history additions.

Disease timing and genotype coding. The baseline disease indicators were I10, E10, E11, I60, I61, I63, I64, G35, G36, G37, G20, G21, G22, G23, G25, G31, G10, G11, G12, F20, F30, F31, F32, and F33, entered separately. A first-report date on or before the initial assessment was coded 1; a later report or no report was coded 0. Date strings and numeric Excel serial dates were parsed. Nonempty unparseable or implausible dates (<1905 or after October 7, 2026) were treated as unknown. Indicators without variation in a fitted sample were omitted, with the fitted model ranks documented in the analysis output. The bulk-genotyping indicator (Field 22418) did not identify APOE ε4 status and was not used as an APOE genotype. SNP-labelled dosage columns were not used to infer carrier status without adequate genotype information. MRI models did not adjust for APOE ε4.

Head size centre and sampling interval. No head-size covariate, intracranial volume, or volumetric scaling factor was available in the MRI analytical dataset. No head-size normalization or adjustment was performed. Imaging-centre information was also unavailable, and the baseline recruitment centre (Field 54, instance 0) was not substituted for the imaging centre. Initial-assessment dates were available, but individual instance-2 imaging dates were available only in the partial linkage used for Table S2. The time from baseline blood sampling to MRI was not included, and baseline age did not represent age at imaging. Neither total brain volume nor head BMD was used as a head-size surrogate. These unadjusted imaging factors limit interpretation and leave residual confounding.

Sample rules and estimation. Available-case Models 1–3 used complete records for the selected outcome, OCN, and that model's covariates. For each outcome, all three models and Model 2 + PRS were additionally fitted in precisely the same participants with complete data for every Model 3 variable. Counts were 7,142, 6,869, and 1,969 for most volumes in available-case Models 1, 2, and 3; corresponding WMH counts were 6,972, 6,707, and 1,926. The matched comparisons therefore used 1,969 participants for each non-WMH outcome and 1,926 for WMH in every specification. Among Model 2 participants, 4,900/6,869 for most volumes and 4,781/6,707 for WMH lacked at least one required PRS, primarily the Enhanced apolipoprotein scores; 39 lacked Standard AD PRS in each outcome group, overlapping the Enhanced-score missingness. No additional participants were excluded for unknown history dates after the PRS restriction. Exact counts and estimates are given in Table S3. The MRI analysis used base R 4.6.1, ordinary least squares, conventional model-based standard errors, t-based 95% confidence intervals, and two-sided coefficient t tests. Nominal MRI P values and BH-adjusted q values across the

12 outcomes are reported in Table S3. The same-participant comparisons address changing sample composition between models but do not eliminate selection bias or establish causation.

Supplementary Methods 2B. Baseline statistical path specification

For each cognitive outcome, two exploratory linear-regression specifications were fitted on the same baseline complete-case sample: the BMD-as-X specification used heel BMD as X and OCN as M; the alternative used OCN as X and heel BMD as M. The selected cognitive test was Y in both specifications. The component model was M ~ X + covariates, and the outcome model was Y ~ X + M + covariates. Coefficient a was obtained from X in the component model; b and c' were obtained from M and X, respectively, in the outcome model. OCN, BMD, and log(x + 1)-transformed cognition were z-standardized within each outcome-specific sample. Covariates were age, sex, BMI, Townsend deprivation index, and university-degree status. Individual-coefficient 95% CIs used HC3 heteroscedasticity-robust standard errors and a normal approximation. The statistical product a x b was recalculated in 1,000 participant bootstrap resamples, with percentile 95% CIs; scaling was fixed to the outcome-specific complete-case sample, and the same resampled participants contributed to both component regressions. No exposure-mediator interaction was fitted. Mediated percentages were not reported. Only the explicit i0 numerical Heel BMD, i0 Olink BGLAP, i0 reaction time, and i0 pairs-matching round-1 fields were used; missing baseline values were not filled from later visits. Baseline fluid intelligence and numeric memory had no overlap with the selected Heel BMD field and were omitted. These specifications describe conditional associations and do not identify natural direct or indirect causal effects.

Supplementary Methods 2C. Baseline participant-characteristic definitions

Main Tables 1–2 describe the full OCN cohort by quartile and the longitudinal cohort by incident all-cause dementia. Clinical histories use the earliest available first-report dates on or before the initial assessment. The broader descriptive groups use the following available ICD-10 fields: Diabetes broad (E10, E11, E12, E13, E14); Hypertension broad (I10, I15); Cerebrovascular (I60, I61, I62, I63, I64, I65, I66, I67, I68, G45, G46); Demyelinating (G35, G36, G37); Neurodegenerative (G20, G21, G22, G23, G25, G31, G32, G10, G11, G12); Organic (F04, F05, F06, F07, F09, G91, G92, G93, G94); Mental (F20, F21, F22, F23, F24, F25, F28, F29, F30, F31, F32, F33, F34, F38, F39, F40, F41, F10, F11, F12, F13, F14, F15, F16, F17, F18, F19). Primary-model diabetes, hypertension, and stroke use E10/E11, I10, and I60–I64, respectively, and are displayed separately. Fields absent from the longitudinal extract were linked by participant ID from the full-cohort dataset at the exact specified measurement visit. Existing missing values were not filled, and no later measurement substituted for a missing baseline value. MRI volumes are later instance-2 measurements. Descriptive diagnosis entries in supplementary cohort comparisons cover all available report dates and are distinct from baseline adjustment variables.

Supplementary figures

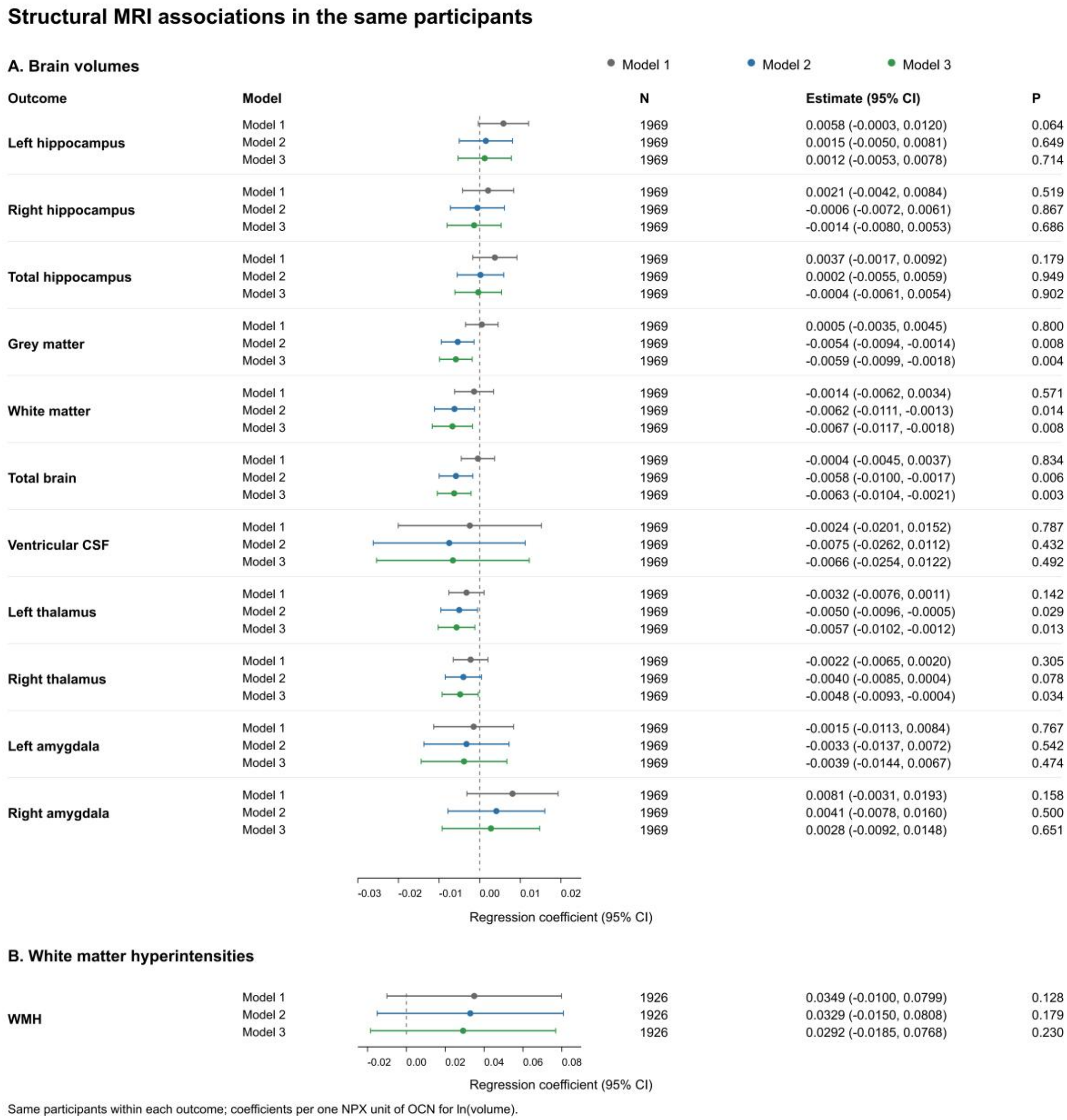

Figure S1. Structural MRI associations across Models 1–3 in identical outcome-specific samples. (A) Eleven regional and global brain-volume outcomes. (B) Total WMH volume. For each outcome, Models 1–3 used the same participants with complete data for all MRI Model 3 variables: N=1,969 for each outcome in panel A and N=1,926 for WMH. Model 1 adjusted for age, age-squared, sex, and BMI; Model 2 additionally included qualifications, Townsend deprivation, smoking, alcohol frequency, moderate physical activity, ethnicity, and handedness. Model 3 additionally included Standard AD PRS, Enhanced apolipoprotein A1/B PRS, and the separate baseline ICD-10 indicators listed in Supplementary Methods 2A. MRI Model 3 did not include APOE ε4 because a validated carrier variable was unavailable. Points and horizontal lines show regression coefficients and 95% CIs per one-unit increase in OCN NPX for ln(volume). Grey, blue, and green identify Models 1, 2, and 3, respectively. The dashed line marks zero. Head size, imaging centre, and the blood-sampling-to-MRI interval were not included. Model 2 + PRS comparisons, available-case estimates, and sample availability are reported in Table S3. MRI P values are nominal. These comparisons do not establish a genetic mechanism. Abbreviations: CI, confidence interval; CSF, cerebrospinal fluid; MRI, magnetic resonance imaging; NPX, Normalized Protein eXpression; OCN, osteocalcin; PRS, polygenic risk score; WMH, white matter hyperintensity. BH-adjusted q values for 12 outcomes within each model/sample family are additionally provided in Table S3; nominal P values remain displayed. MRI model labels 1–3 correspond to M1–M3 and differ from cognitive C1–C3.

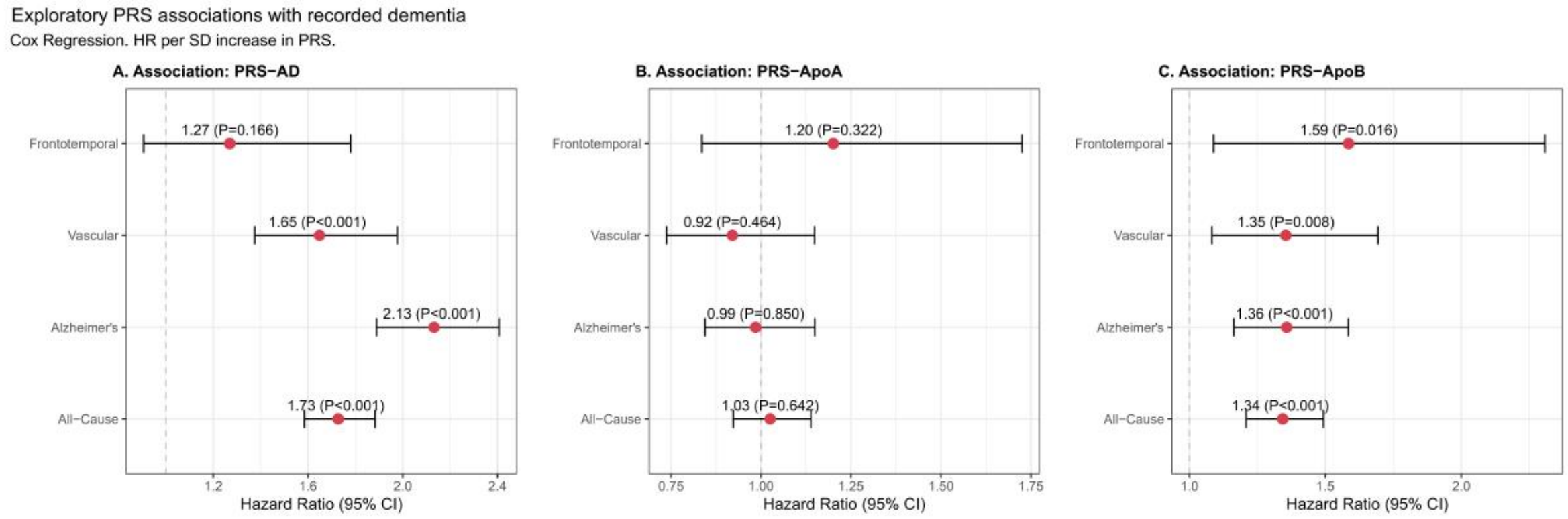

Figure S2. Exploratory PRS associations with recorded dementia outcomes. Hazard ratios (HRs) are per SD increase in PRS. The panels compare AD, apolipoprotein A1, and apolipoprotein B genetic scores across recorded dementia outcomes. Points and horizontal lines show HRs and 95% CIs; dashed vertical lines mark HR=1. Complete covariate and administrative censoring details were unavailable for these comparisons, which are interpreted descriptively rather than as fully adjusted primary estimates. They do not validate clinical prediction. Abbreviations: AD, Alzheimer's disease; CI, confidence interval; HR, hazard ratio; PRS, polygenic risk score; SD, standard deviation.

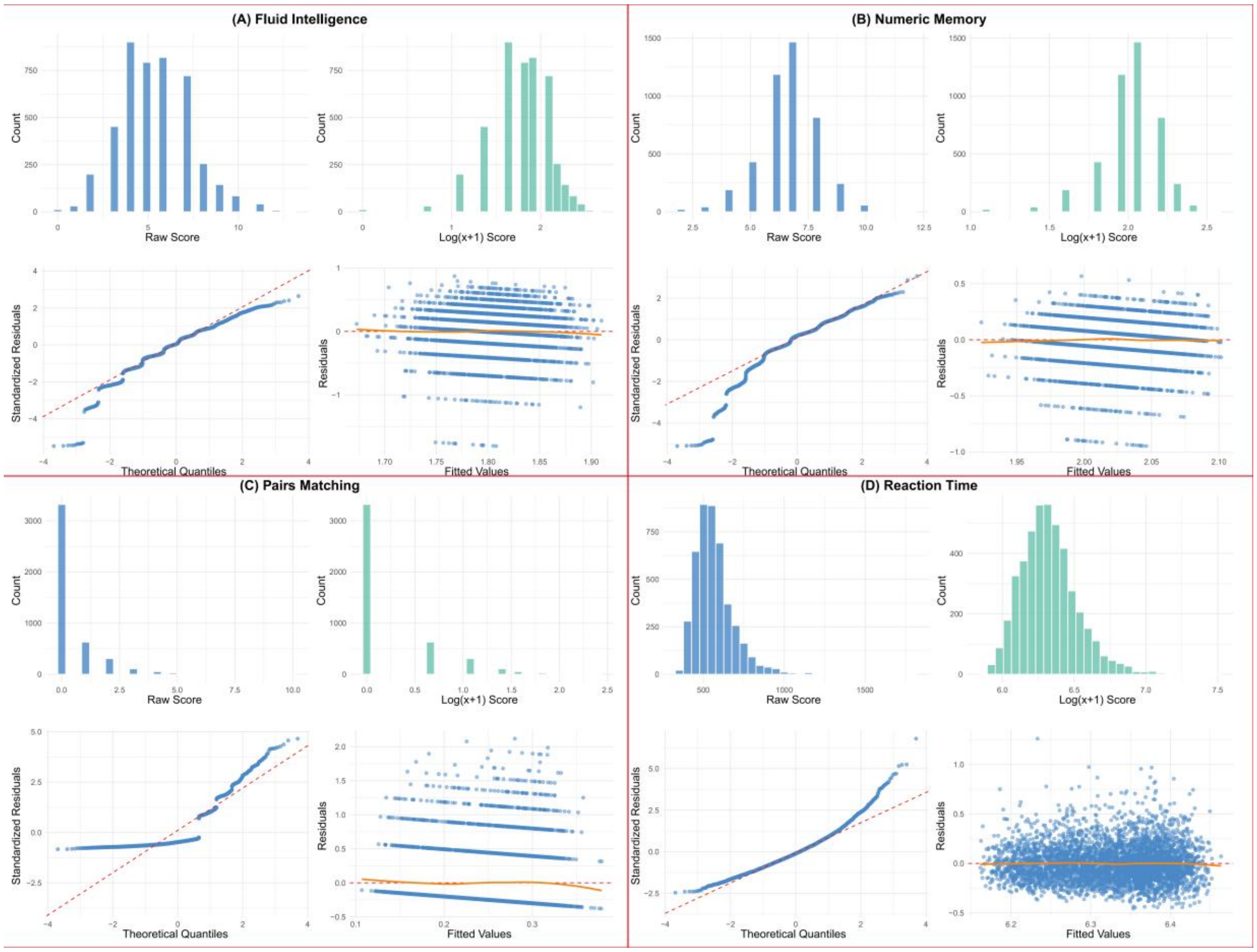

Figure S3. Distribution and regression diagnostics for four cognitive domains. Panels A-D show fluid intelligence, numeric memory, pairs-matching errors, and reaction time, respectively. Each domain includes the raw distribution, log(x+1) distribution, a normal Q-Q plot of standardized residuals, and residuals against fitted values from the exploratory diagnostic regression. These plots inspect distribution and model fit rather than establish normality or causal validity. Pairs matching remains zero-inflated and heteroscedastic after transformation, motivating the Negative Binomial sensitivity results in Table S4B. Longer reaction times and more errors indicate poorer performance. Samples are outcome-specific; raw and transformed summaries are separated in the baseline tables. Abbreviations: Q-Q, quantile-quantile. These exploratory diagnostics do not establish the residual distribution of Models C1–C3.

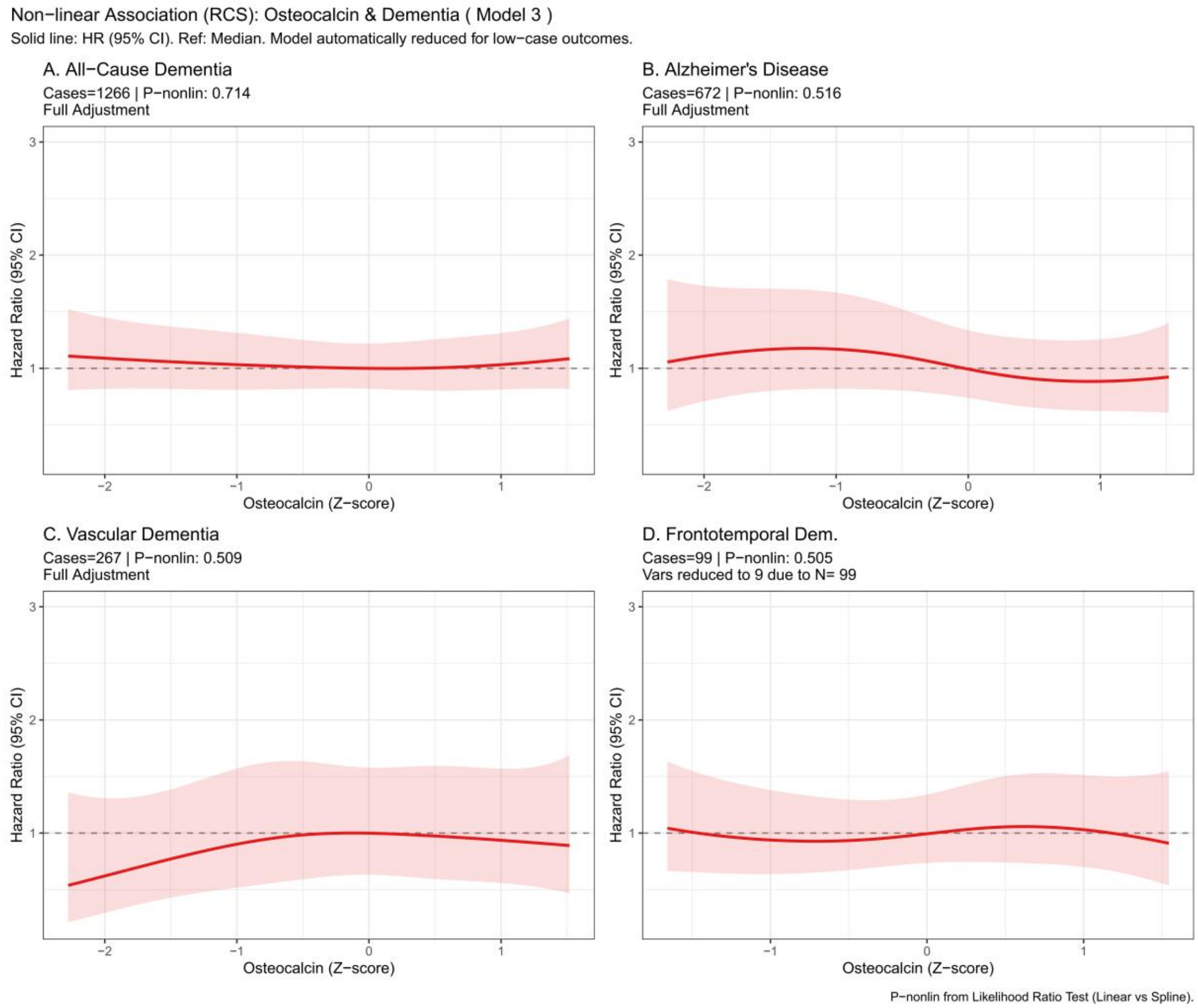

Figure S4. Exploratory OCN–dementia spline models. Panels A–D show all-cause dementia, AD, VaD, and FTD, respectively. Curves and shaded regions show HRs and 95% CIs, with HR=1 indicated by a dashed line and median standardized OCN as reference. Nonlinearity P values are displayed. The FTD specification was reduced because of sparse events and used 99 events. These spline models differ from the primary continuous Model 3 specifications; outcome annotations do not define the complete-record primary event counts. Continuous primary estimates are reported in Table S11. Abbreviations: AD, Alzheimer's disease; CI, confidence interval; FTD, frontotemporal dementia; HR, hazard ratio; OCN, osteocalcin; VaD, vascular dementia.

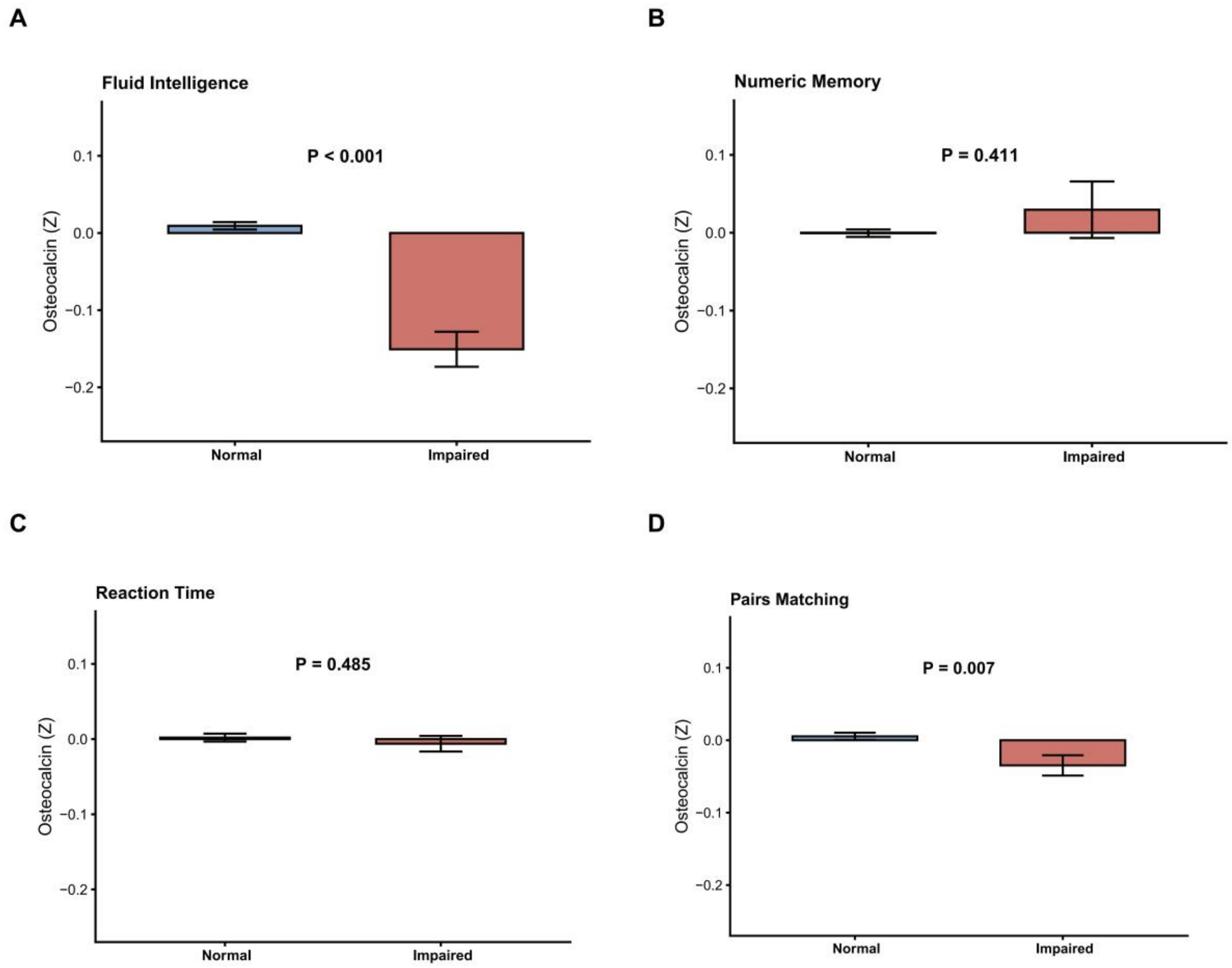

Figure S5. Relative OCN across cognitive performance groupings. Panels A-D compare standardized Olink BGLAP values in the normal and worst-performing quartile groups for fluid intelligence, numeric memory, reaction time, and pairs matching, respectively. For fluid intelligence and numeric memory, the worst quartile contains the lowest scores; for reaction time and pairs matching, it contains the longest times or most errors. Distributions and nominal unadjusted P values are shown. These relative NPX group comparisons do not establish neuroprotection or clinical predictive utility. Cognitive scores and diagnosis-group comparisons are described in the participant-characteristic tables. Abbreviations: NPX, Normalized Protein eXpression; OCN, osteocalcin.

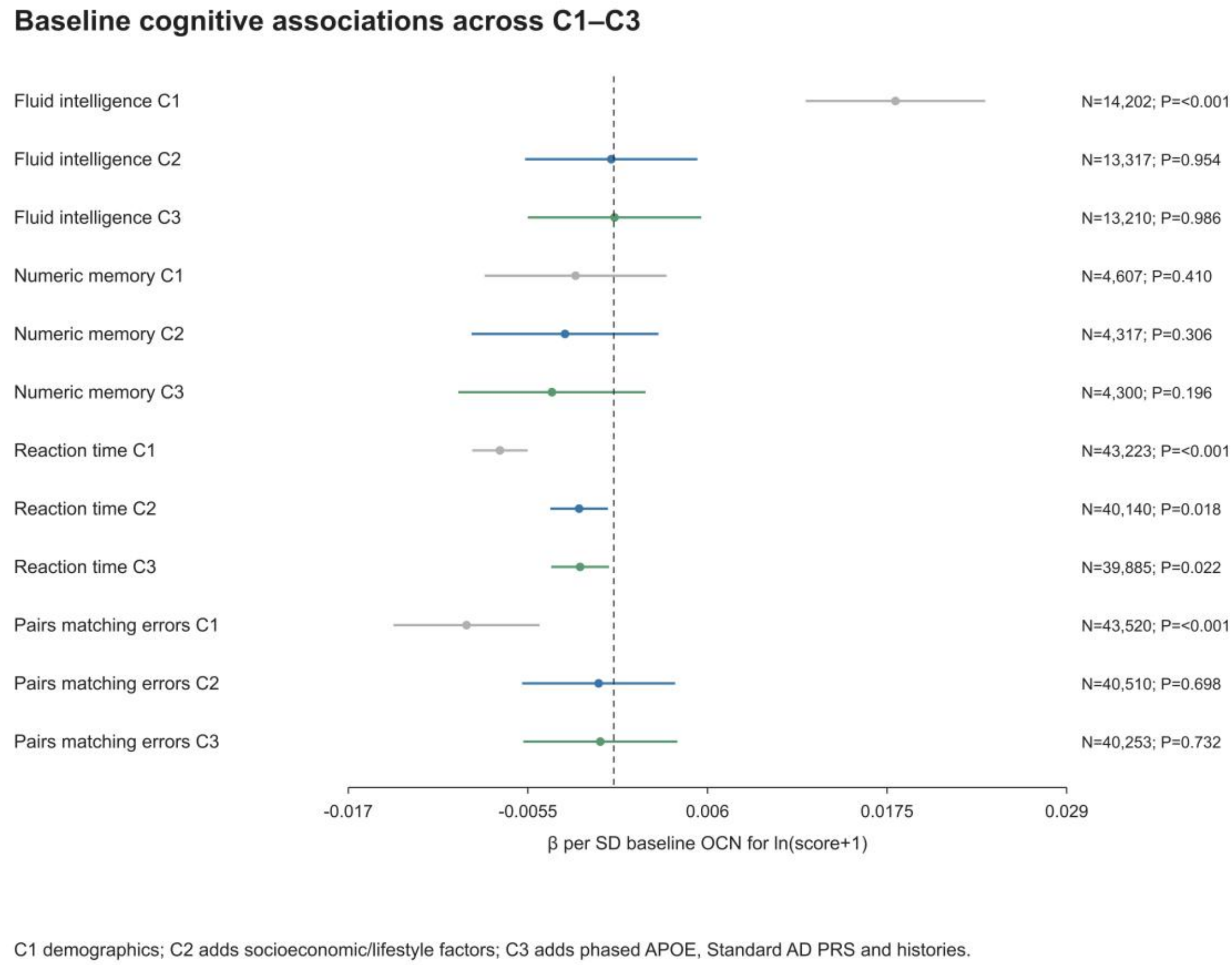

Figure S6. Baseline cognitive associations in Models C1–C3. The prospective extract, instance-0 fields, ln(score+1) transformation and per-SD OCN scale match Figure 2A and Table S4. C1 includes age, age-squared, sex and BMI; C2 adds education, Townsend, smoking, alcohol, activity and ethnicity; C3 adds Standard AD PRS, phased APOE ε4 and baseline hypertension, diabetes and stroke. Gray, blue and green distinguish C1, C2 and C3. Dots and horizontal lines show coefficients and 95% CIs; dashed lines mark zero. Available-case N can differ between models.

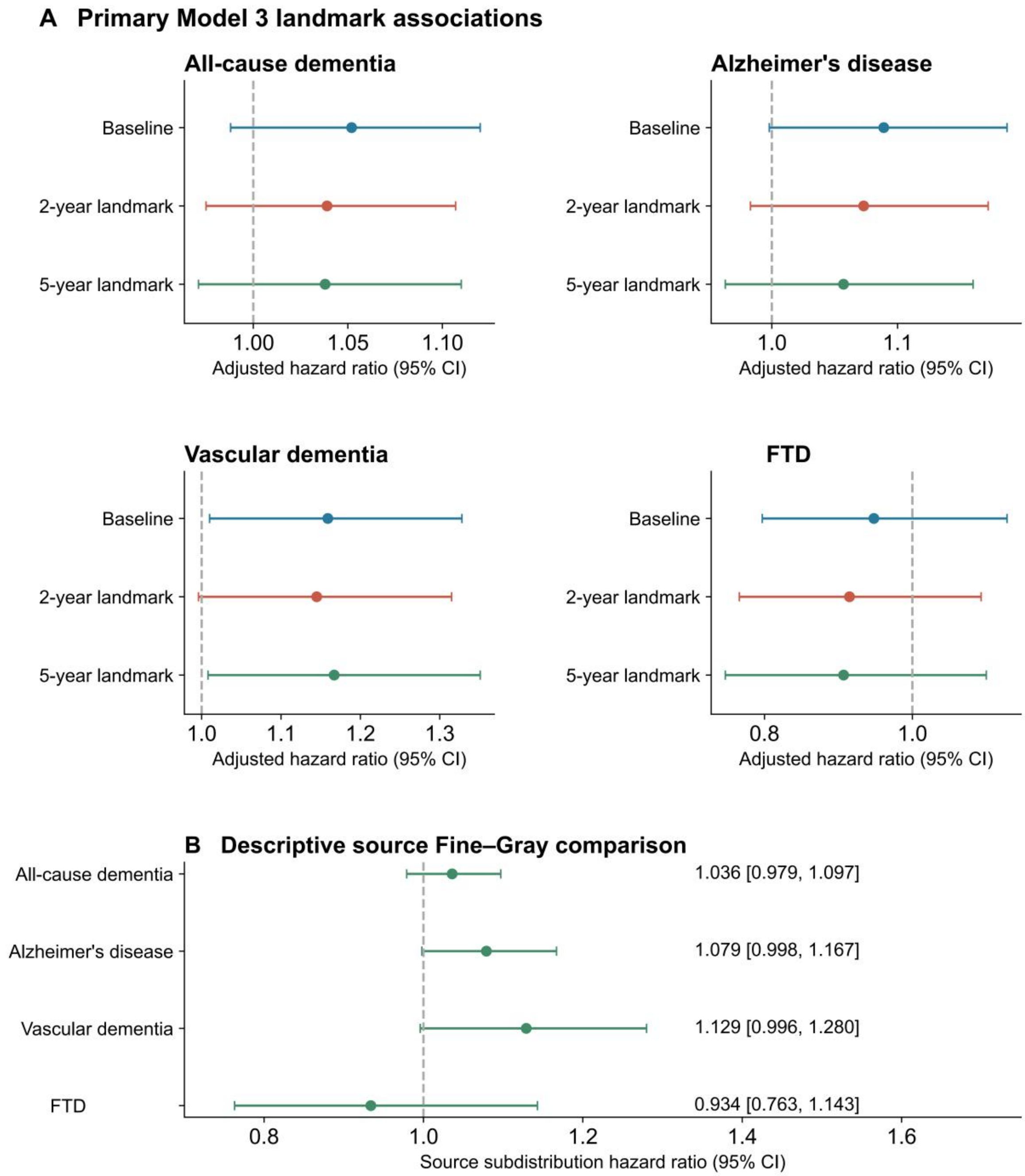

Figure S7. Landmark and descriptive competing-risk sensitivity comparisons. (A) OCN HRs for four outcomes at baseline and after 2- and 5-year landmarks, using primary Model 3 covariates. Participants at each landmark were alive, uncensored, and free of recorded dementia, and follow-up began at that landmark. Baseline OCN scaling, outcome definitions, and the November 27, 2024 cutoff were maintained. N, events, and P values are in Table S15. Subtype comparisons remain exploratory. (B) Fine–Gray subdistribution HRs with death as the competing event, from separate exploratory models adjusted for age, age-squared, sex, and BMI. These minimally adjusted comparisons use a different specification from Model 3. Points and horizontal lines show estimates and 95% CIs; dashed vertical lines mark HR=1. Abbreviations: BMI, body mass index; CI, confidence interval; FTD, frontotemporal dementia; OCN, osteocalcin.

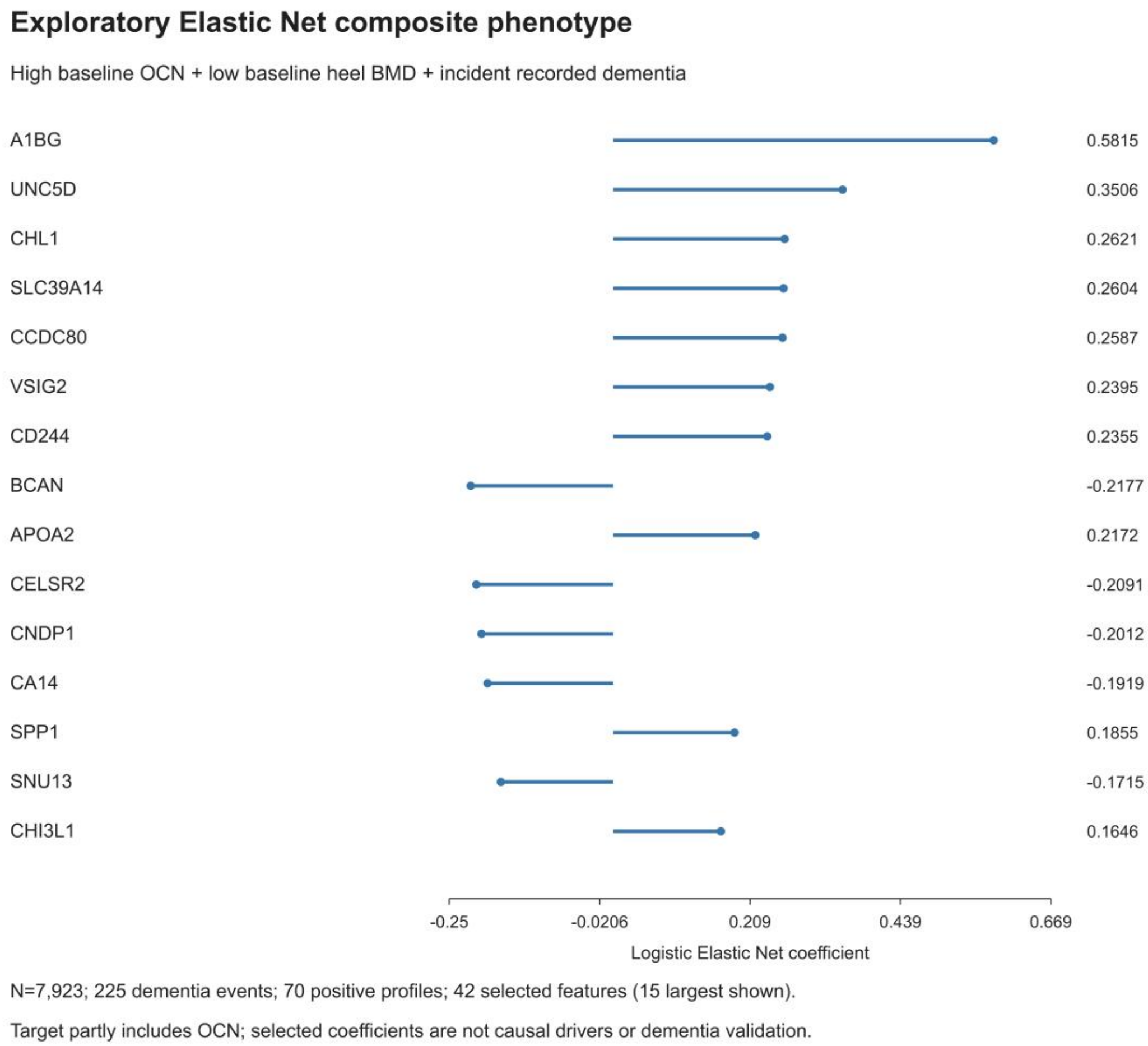

Figure S8. Elastic Net coefficients for the baseline OCN–BMD–dementia composite profile. Positive profile requires OCN above the prospective-cohort median, baseline heel BMD below its observed median, and incident dementia before censoring. The measured complete matrix has 7,923 participants, 225 dementia events and 70 positive profiles. Binomial glmnet used alpha=0.5, standardized predictors, ten target-stratified folds, AUC criterion, seed 20261007 and lambda.min=0.004084. Fifteen largest absolute nonzero coefficients are shown; all 42 selected coefficients and lambda.1se are in Table S10. The composite is partly defined by OCN and cannot establish dementia-specific prediction or causal drivers. These features did not enter the pathway foreground.

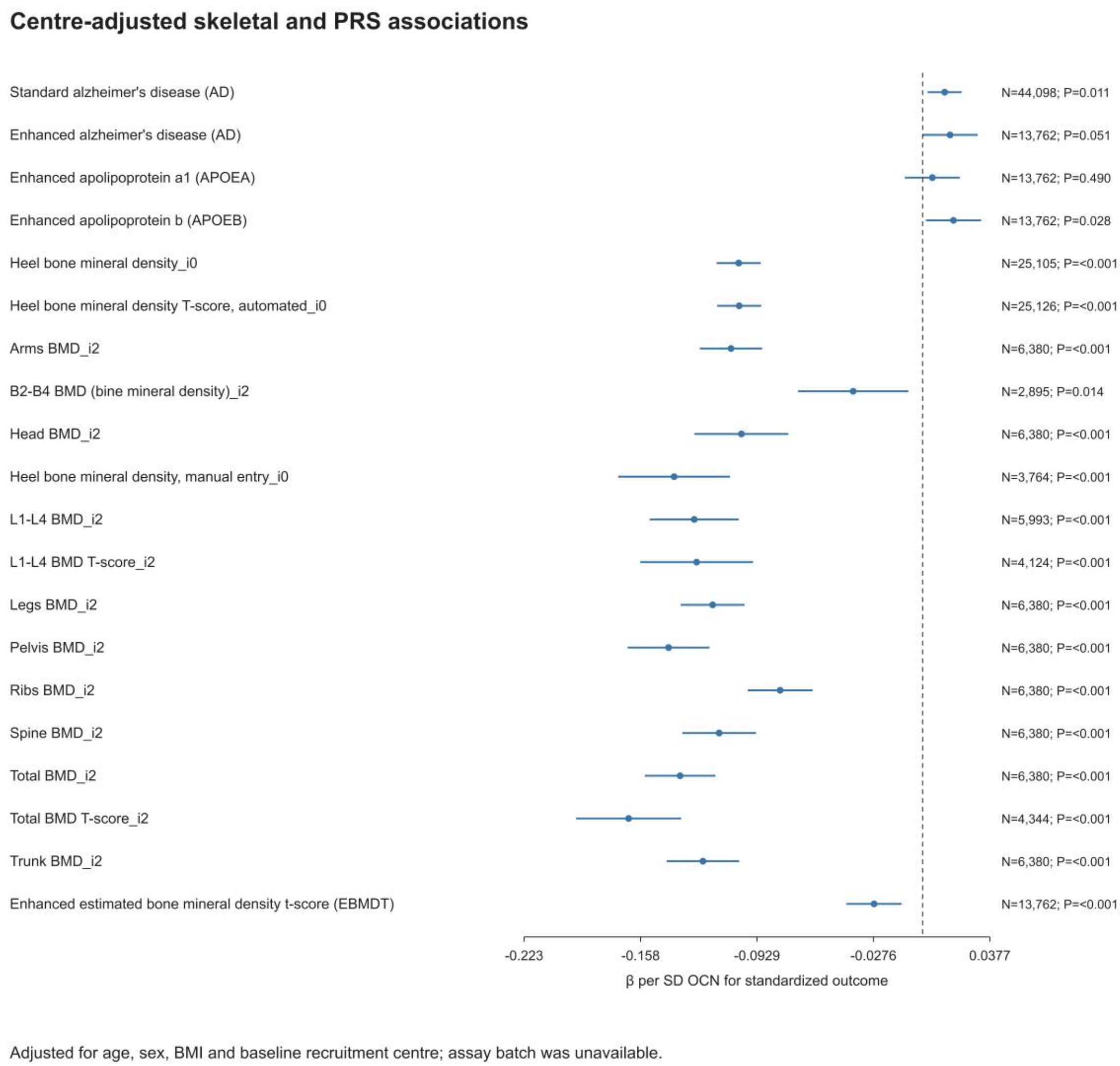

Figure S9. Skeletal and PRS associations with baseline OCN. OCN and each outcome were standardized; regressions adjusted for baseline age, sex, BMI and recruitment centre. Assay batch was unavailable. Instance-0 heel measures and later instance-2 DXA phenotypes are explicitly distinguished; PRS field names distinguish Standard AD, Enhanced AD, Enhanced apolipoprotein A1/B and Enhanced estimated-BMD scores. Dots and horizontal lines show coefficients and 95% CIs per SD OCN for standardized outcomes; nominal P and N are printed. Full estimates are in Table S24.

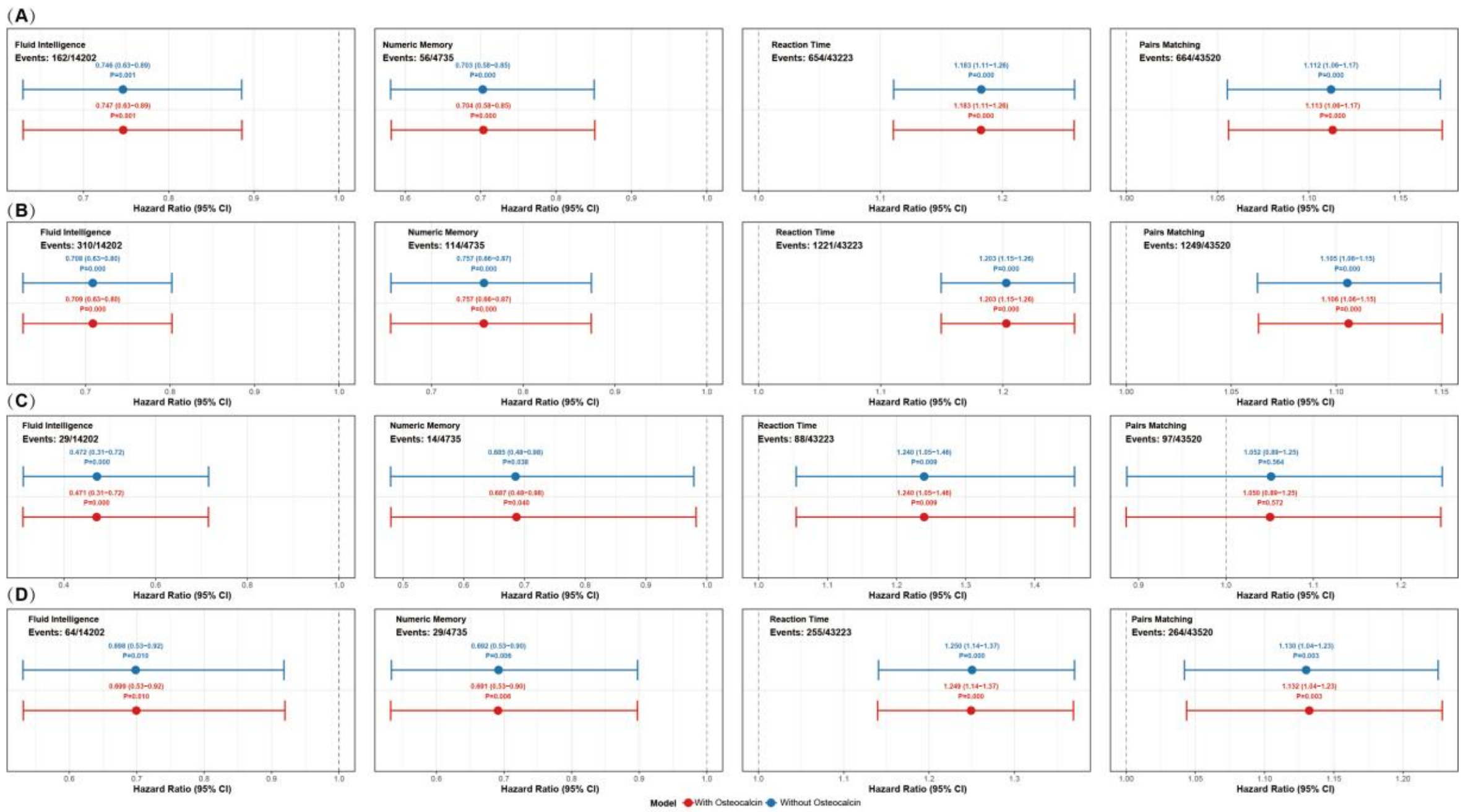

Figure S10. Exploratory cognitive-dementia Cox associations with and without additional OCN adjustment. Panels A-D show estimates for all-cause dementia, Alzheimer's disease, FTD, and vascular dementia, respectively. Within each panel, fluid intelligence, numeric memory, reaction time, and pairs matching are compared. Colors distinguish inclusion versus omission of OCN; horizontal lines indicate 95% confidence intervals, and vertical dashed lines indicate HR=1. The event/sample counts for fluid intelligence, numeric memory, reaction time, and pairs matching were 310/14,202, 114/4,735, 1,221/43,223, and 1,249/43,520 for all-cause dementia; 162/14,202, 56/4,735, 654/43,223, and 664/43,520 for AD; 29/14,202, 14/4,735, 88/43,223, and 97/43,520 for FTD; and 64/14,202, 29/4,735, 255/43,223, and 264/43,520 for vascular dementia. Blue denotes omission of OCN and red its inclusion. The complete covariate and administrative censoring details were unavailable for these exploratory models, which use a different specification from primary Model 3. Adding OCN is an adjustment comparison rather than a causal mediation test. Higher reaction time and pairs-matching errors denote poorer performance. Abbreviations: CI, confidence interval; FTD, frontotemporal dementia; HR, hazard ratio; OCN, osteocalcin.

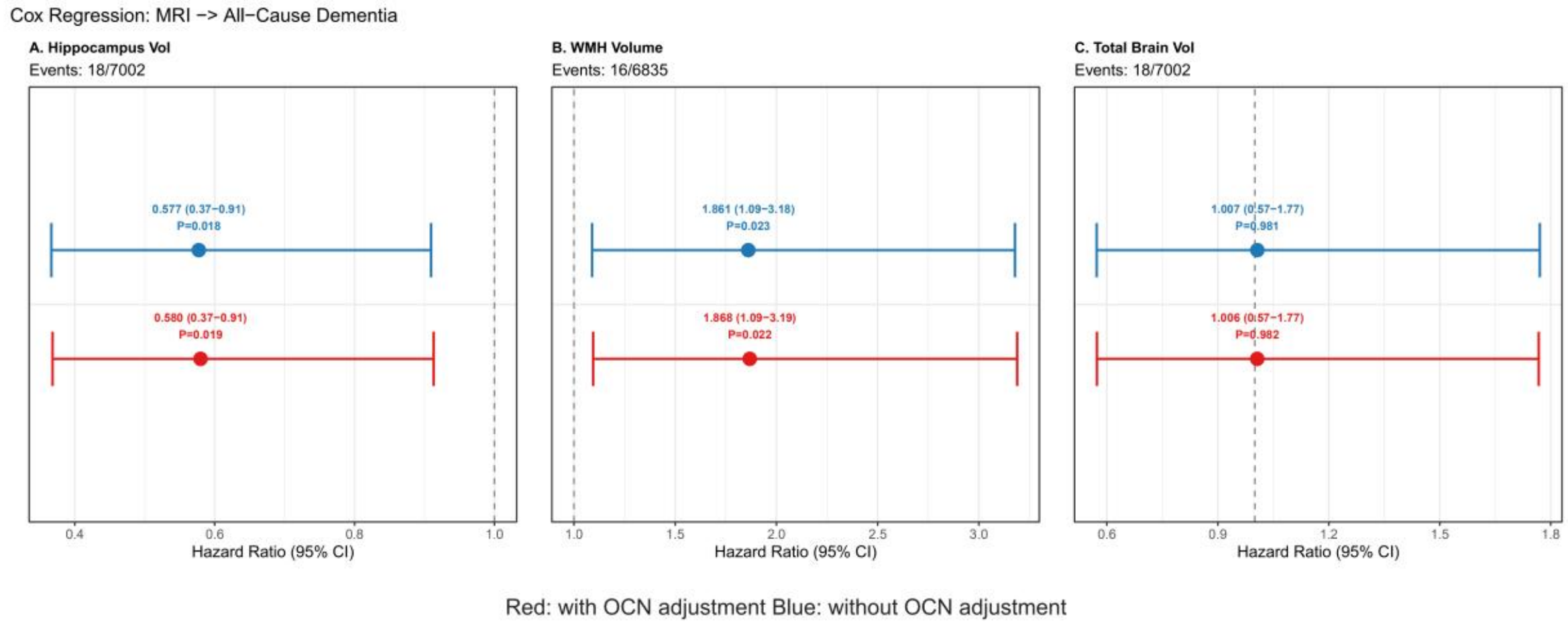

Figure S11. Exploratory Cox associations of MRI phenotypes with all-cause dementia, with and without OCN adjustment. The three component plots show hippocampal, WMH, and total brain volumes; colors identify OCN-adjusted versus unadjusted-for-OCN estimates. Points indicate hazard ratios and horizontal lines their 95% confidence intervals; the dashed vertical line marks HR=1. Component plots provide the event and sample counts. MRI was measured at later visits, and the sequence relative to first recorded diagnosis could not be confirmed throughout the sample. Complete covariate and administrative censoring details were unavailable for these exploratory models. These comparisons are descriptive and cannot establish temporal or causal mediation. Abbreviations: CI, confidence interval; HR, hazard ratio; MRI, magnetic resonance imaging; OCN, osteocalcin; WMH, white matter hyperintensity.

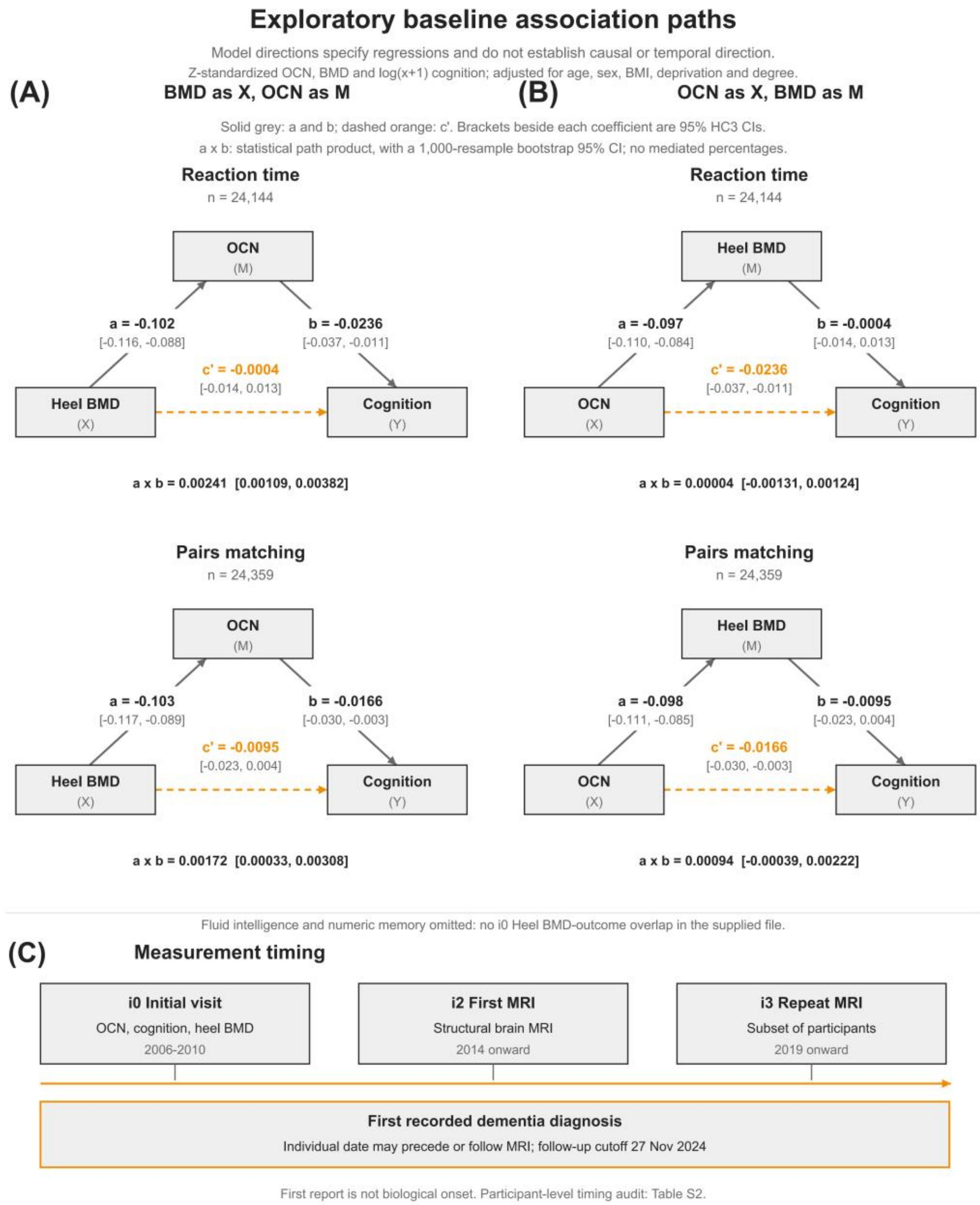

Figure S12. Exploratory baseline association paths and measurement timing. (A) Heel BMD as exposure X and relative OCN as intermediate variable M. (B) Relative OCN as X and heel BMD as M. Each panel presents reaction time and pairs-matching errors separately, using identical participants for the two specifications within each outcome (n=24,144 and n=24,359, respectively). Solid grey arrows denote regression coefficients a and b; dashed orange arrows denote the conditional coefficient c'. Brackets show 95% HC3 confidence intervals. The statistical product a x b is accompanied by its percentile 95% CI from 1,000 participant bootstrap resamples. OCN, BMD and log(x+1)-transformed cognition were z-standardized within each complete-record sample. Models adjusted for age, sex, BMI, Townsend deprivation and university-degree status. No exposure-intermediate-variable interaction was fitted. Concurrent coefficients and products do not establish causal mediation, and no mediated percentages are reported. Fluid intelligence and numeric memory are omitted because their baseline fields did not overlap the selected numerical heel BMD field. (C) Measurement timing: OCN, cognition and heel BMD were recorded at the initial visit, whereas MRI was obtained at later visits. First recorded dementia dates can precede or follow imaging; the partial participant-level audit is reported in Table S2. First report is not biological onset. Abbreviations: BMD, bone mineral density; CI, confidence interval; HC3, heteroscedasticity-robust standard error correction; MRI, magnetic resonance imaging; OCN, osteocalcin.

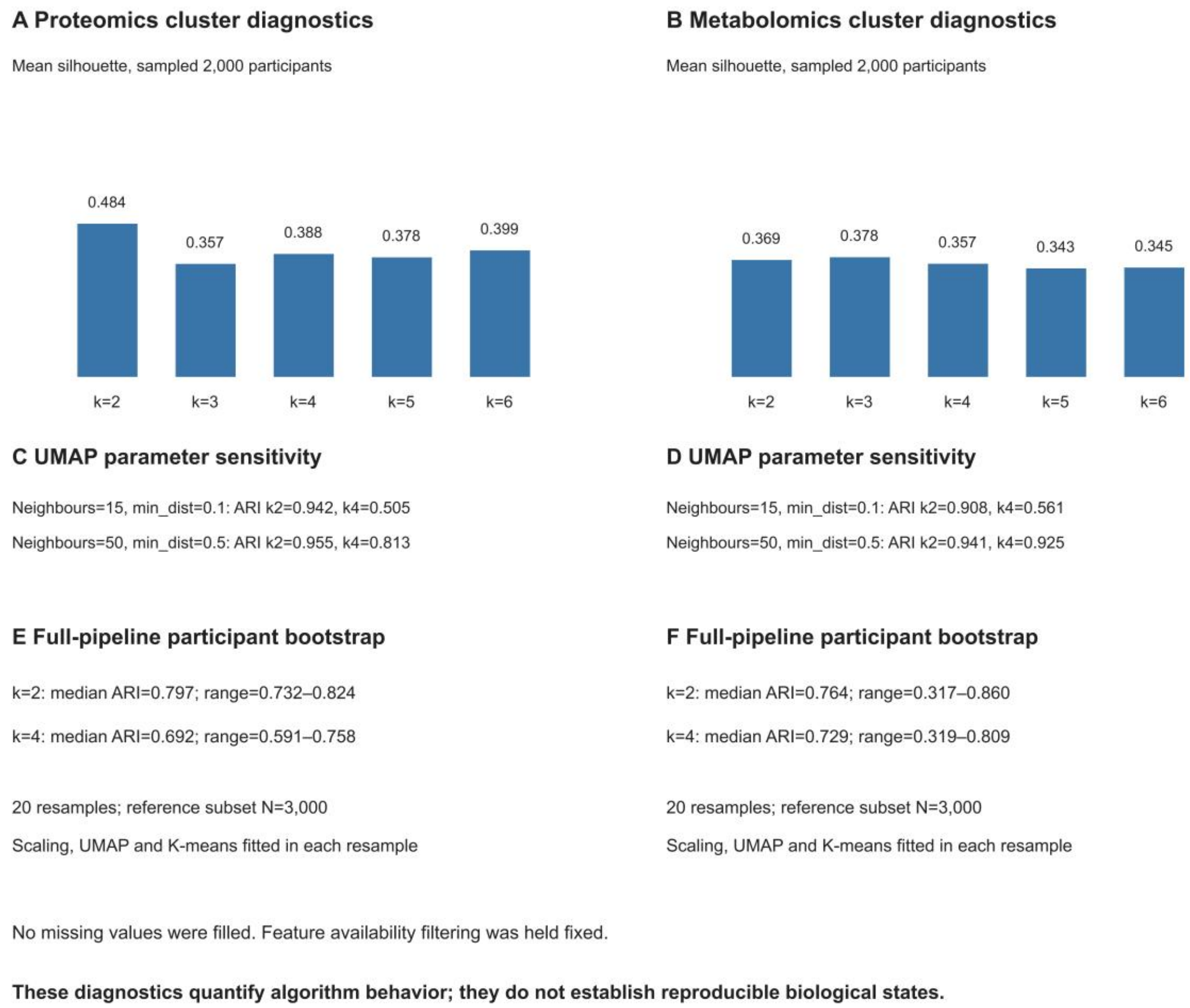

Figure S13. Measured-only clustering diagnostics. (A–B) Mean silhouette for k=2–6, evaluated in 2,000 sampled complete participants. (C–D) ARI relative to the full-matrix 30-neighbour/min_dist=0.3 solution when scaling, UMAP and K-means were fitted with 15/0.1 or 50/0.5. (E–F) Median and range of ARI across 20 participant bootstrap resamples in a 3,000-person evaluation subset, with scaling, UMAP and Lloyd K-means fitted in each resample. Unique resampled IDs were compared with the subset reference. Availability-filtered feature sets were held fixed. No missing values were filled. These are conditional algorithm diagnostics, not evidence of reproducible biological states. Table S28 reports k=2 and k=4 composition results and all diagnostics.

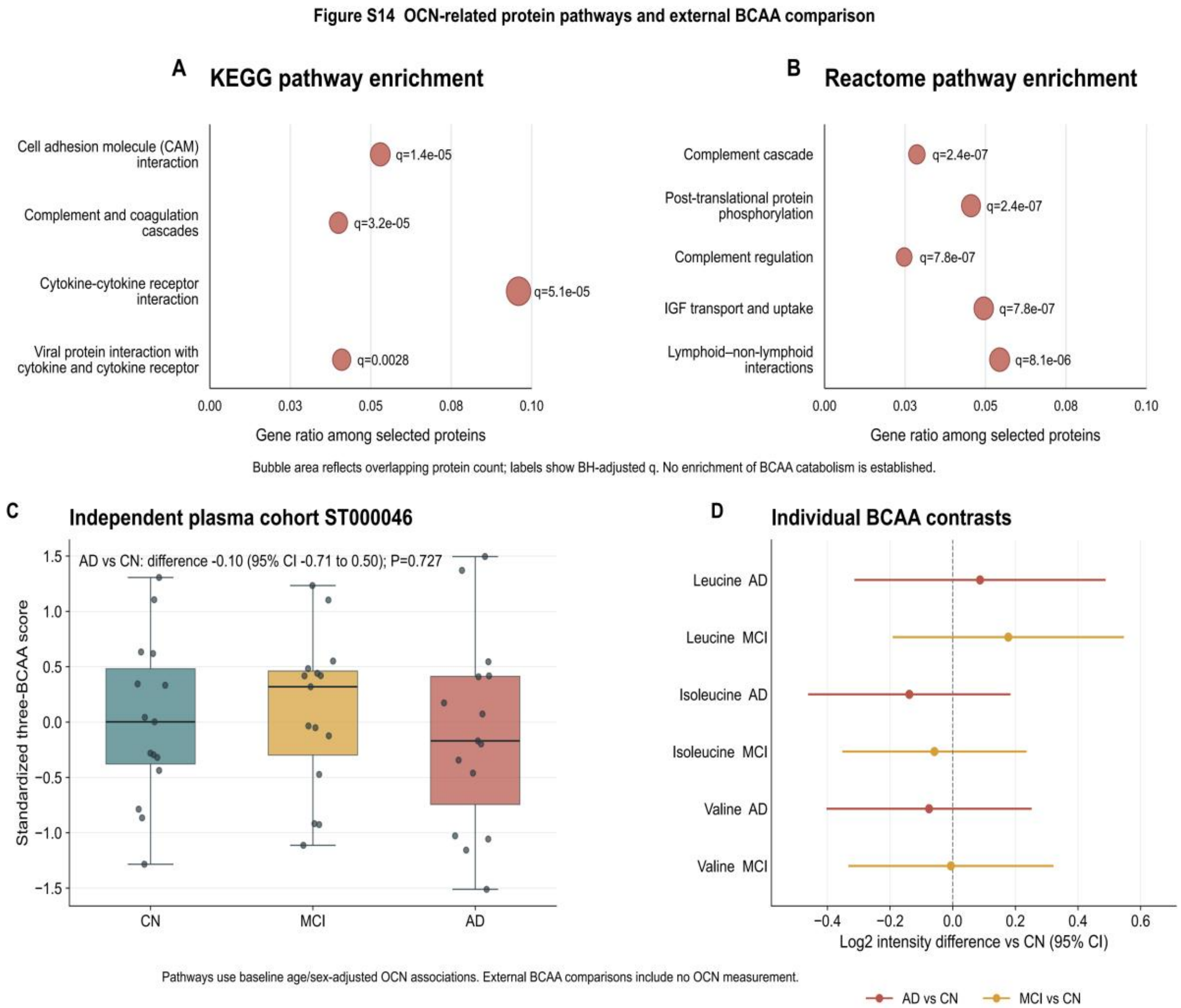

Figure S14. OCN-related protein pathways and independent BCAA comparison. (A–B) KEGG and Reactome over-representation results using baseline observed proteins that survived age- and sex-adjusted panel-wise Bonferroni correction. No Elastic Net-selected features were added. The measured assay background contains 2,911 mapped symbols (2,872 KEGG Entrez IDs); the foreground contains 1,011 symbols (1,001 KEGG IDs). Bubble position shows the fraction of the foreground overlapping each pathway, bubble area reflects the overlapping protein count, and labels give BH-adjusted q values. Every eligible pathway with 5–500 measured-background genes was included in correction across 321 KEGG and 1,130 Reactome terms. The displays show the leading corrected terms and do not establish vascular injury or enrichment of BCAA catabolism. (C) External ST000046 observations for 15 cognitively normal (CN), 15 mild cognitive impairment (MCI), and 15 AD participants, with the standardized three-BCAA score shown as a box plot and individual observations. The AD–CN score difference is −0.104 (95% CI, −0.709 to 0.501; P=0.727). (D) Individual leucine, isoleucine, and valine contrasts for AD–CN and MCI–CN, with points and 95% CIs; colors identify the two comparisons. Log2 LC–MS intensities are relative signals, and the cohort did not measure OCN. BH correction covers the three individual BCAAs within each comparison, with a separate two-contrast correction for the composite score, as reported in Table S30. The external comparison does not directly validate OCN–BCAA coupling. Abbreviations: BCAA, branched-chain amino acid; BH, Benjamini–Hochberg; CI, confidence interval; KEGG, Kyoto Encyclopedia of Genes and Genomes.
